# Crotonylation of MCM6 enhances chemotherapeutics sensitivity of breast cancer via inducing DNA replication stress

**DOI:** 10.1111/cpr.13759

**Published:** 2024-10-30

**Authors:** Haoyun Song, Zhao Guo, Kun Xie, Xiangwen Liu, Xuguang Yang, Rong Shen, Degui Wang

**Affiliations:** ^1^ School of Basic Medical Sciences Lanzhou University Gansu China; ^2^ NHC Key Laboratory of Diagnosis and Therapy of Gastrointestinal Tumor Lanzhou Gansu Province China

## Abstract

Breast cancer is associated with high morbidity and mortality, which are closely influenced by protein post‐translational modifications (PTMs). Lysine crotonylation (Kcr) serves as a newly identified PTM type that plays a role in various biological processes; however, its involvement in breast cancer progression remains unclear. Minichromosome maintenance 6 (MCM6) is a critical component of DNA replication and has been previous confirmed to exhibit a significant role in tumorigenesis. Despite this, a comprehensive analysis of MCM6, particularly regarding its modifications in breast cancer is lacking. In this study, we found MCM6 is upregulated in breast invasive carcinoma (BRCA) and is associated with poorer overall survival by regulating the DNA damage repair mechanisms. Furthermore, MCM6‐knockdown resulted in decreased cell proliferation and inhibited the DNA replication, leading to DNA replication stress and sustained DNA damage, thereby enhancing the chemotherapeutic sensitivity of breast cancer. Additionally, SIRT7‐mediated crotonylation of MCM6 at K599 (MCM6‐K599cr) was significantly upregulated in response to DNA replication stress, primarily due to the disassemebly of the MCM2‐7 complex and regulated by RNF8‐mediated ubiquitination. Concurrently, kaempferol, which acts as a regulator of SIRT7, was found to enhance the Kcr level of MCM6, reducing tumour weight, particular when combined with paclitaxel, highlighting its potential chemotherapeutic target for BRCA therapy.

## Graphical abstract

SIRT7‐mediated crotonylation (Kr) of minichromosome maintenance 6 (MCM6) induced DNA replication stress and regulated by RNF8‐mediated ubiquitination. Kaempferol, which acted as a regulator of SIRT7, could enhance MCM6 Kcr level, alleviating tumour weight, especially combined with paclitaxel, which presents a potential chemotherapeutic target for BRCA therapy.

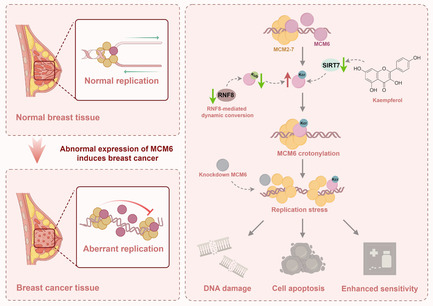

## 1 INTRODUCTION

Breast cancer poses a significant threat to women's health globally. According to the World Cancer Statistics 2023, breast cancer is the most prevalent type of malignancy and the second leading cause of cancer‐related mortality among females.^1^ DNA damage‐targeting chemotherapy is frequently utilized in the treatment of breast cancer. However, the effectiveness of these treatment is often compromised by chemoresistance, which is primarily due to an enhanced capacity for DNA damage repair.^2^, ^3^, ^4^ The DNA damage response (DDR) system is crucial for maintaining genomic integrity and fidelity, and deficiencies in DNA damage repair can result in severe genome instability.^5^, ^6^ Consequently, inducing DDR deficiency in tumour cells has emerged as a promising strategy to overcome chemotherapeutic resistance and improve the efficacy of breast cancer treatment.^7^, ^8^

Replication stress is a driving force of tumorigenesis, which can be targeted for therapeutic intervention.^9^, ^10^ Cells encounter numerous obstacles, both intracellular and extracellular, many of which induce replication stress, leading to aberrant and stalled DNA replication forks, as well as severe DNA damage.^11^ Minichromosome maintenance 6 (MCM6), a vital component of the MCM2‐7 complex, serves as a DNA replicative helicase that is recruited to replication origins during the initiation phase of DNA replication.^12^ Alongside with other MCM subunits, MCM6 collaborates to facilitate the unwinding process and ensure accurate genome replication, as a significant marker of multiple cancer types. However, deficiency of MCM6 affects the stability of the MCM2‐7 complex, thereby suppressing the routine DNA replication and inducing DNA replication stress. Yu et al.,^13^ observed that knockdown of MCM6 hindered the accumulation of MDC1 at DNA damage sites and disrupted the DNA damage repair in oesophageal squamous cell carcinoma (ESCC) cells. Li et al.,^14^ demonstrated that the absence of MCM6 inhibited phosphorylation of CHK2 and affected activation of the ATM‐CHK2 pathway in oral squamous cell carcinoma (OSCC) cells. Thus, MCM6 not only plays a crucial role in regulating DNA replication but also contributes to tumorigenesis by modulating DNA damage repair. Despite more extensive research on other members of the MCM family proteins, investigation into MCM6 is still in its early stages.

Deletion and post‐translational modifications (PTMs) of MCM6 are associated with human diseases, particularly in cancer, also as profound impacts on therapy resistance. Previous studies verified that knockdown of MCM6 inhibited various tumour cells proliferation and indicated its potential role in tumour progression, including brain,^15^ lymphomas,^16^ lung^17^ and prostate cancer.^18^ RNF125‐mediated ubiquitination of MCM6 promoted its degradation, thus impeded the proliferation of hepatocellular carcinama (HCC) cells.^19^ Meanwhile, MCM6 interacted with UBE3A and enhanced ubiquitination of p53, mediating HCC malignant behaviours.^20^ However, little is known PTMs of MCM6 in breast cancer.

The discovery of lysine crotonylation (Kcr), a highly evolutionarily conserved PTM, marks a significant advance in our understanding of gene regulation, spermatogenesis, metabolism and disease progression.^21^, ^22^, ^23^ Since the emerging role of Kcr in regulating gene expression, histone Kcr in DNA damage repair also existed.^24^, ^25^ Abu‐Zhayia et al., revealed that H3K9cr showed rapid reduction upon exposure to ionising radiation (IR), ultraviolet (UV), or the damaging agent VP16, which indicated that the recovery of H3K9cr after exposure to genotoxic agents influence DNA damage.^26^ With the advancement of modern mass spectrometry (MS) technology, several proteomics studies have expanded Kcr substrates to non‐histone proteins.^27^, ^28^ The activation of MTHFD1‐Kcr promotes the development of pancreatic cancer by increasing resistance to ferroptosis.^29^ However, the functional impact of non‐histone Kcr remains to be explored, especially in regulating DNA replication stress.

This study has identified that high expression levels of MCM6 may serve as an effective early diagnostic and prognostic biomarker for breast cancer. Knockdown of MCM6 resulted in DNA replication stress and endogenous DNA damage, which induced breast cancer cells apoptosis and increased their sensitivity to chemotherapeutic agents. Additionally, SIRT7‐mediated MCM6‐Kcr induced DNA replication stress and led to the accumulation of DNA damage by affecting the stability of the MCM2‐7 complex, thereby further enhancing sensitivity to chemotherapeutic drugs. Furthermore, the natural product kaempferol inhibited SIRT7 expression and upregulated the MCM6‐Kcr levels, effectively improving the sensitivity of breast cancer cells to chemotherapeutic treatments.

## 2 RESULTS

### 2.1 Pan‐cancer analysis of MCM6 expression

**FIGURE 1:**
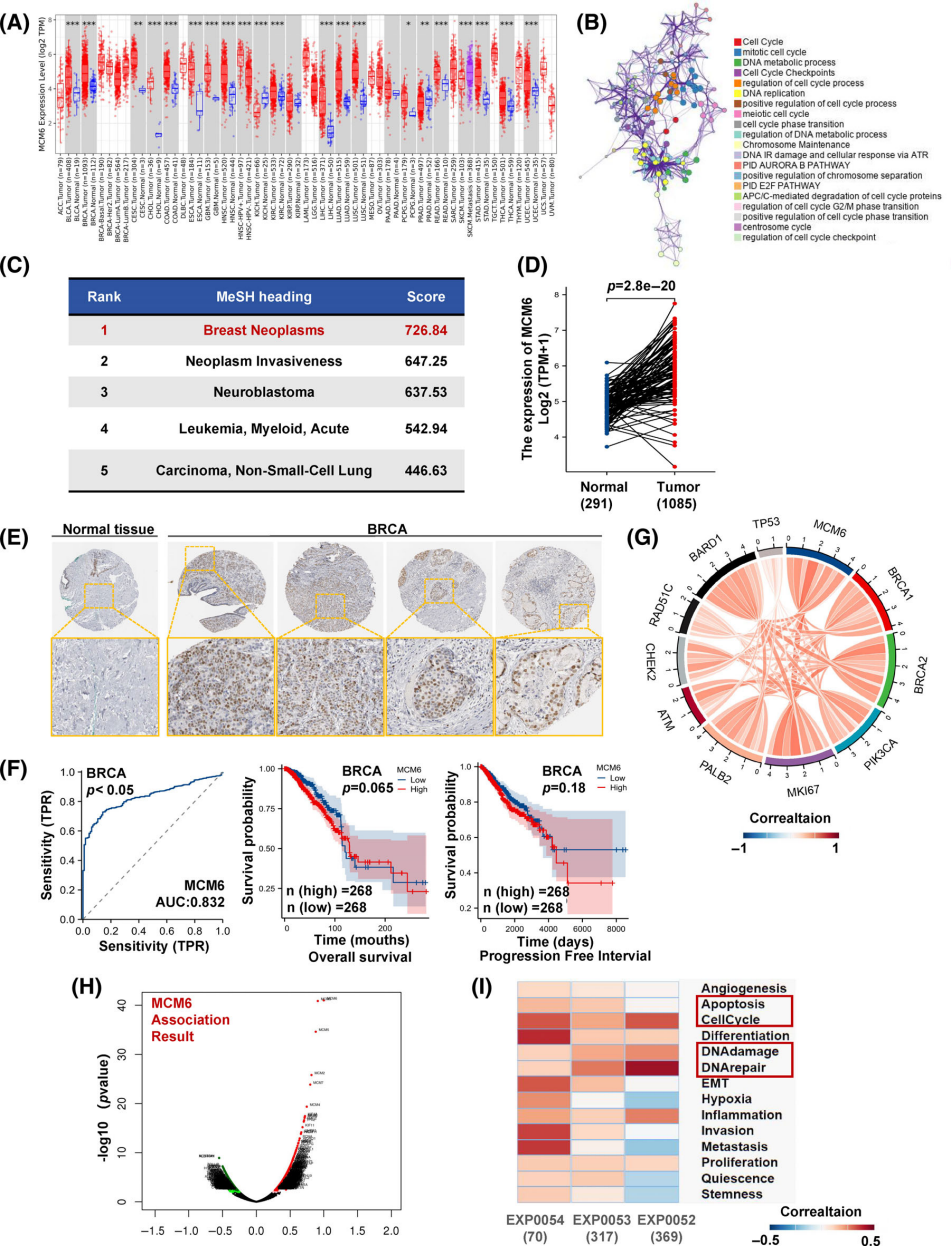
High expression of minichromosome maintenance 6 (MCM6) has clinical value in breast invasive carcinoma (BRCA). (A) The expression of MCM6 in the TIMER 2.0 database. (B) The Kyoto Encyclopedia of Genes and Genomes and Genome Ontology (KEGG/GO) enrichment based on MCM6 binding proteins using Metascape database. (C) Form of associated diseases based on Coexpedia. (D) Comparison of MCM6 mRNA expression levels according to Gene Expression Profiling Interactive Analysis, version 2 (GEPIA 2.0). (E) Immunohistochemistry images of MCM6 in BRCA and normal tissues detected in the human protein atlas database. (F) Receiver operating characteristic curves of highly MCM6‐associated tumours predicated in BRCA; Kaplan–Meier survival curves of overall survival and progressive free interval in BRCA. (G) Circos plot displaying the interconnectivity between MCM6 and the breast cancer biomarkers BRCA1, BRCA2, PIK3CA, MKI67, PALB2, ATM, CHEK2, RAD51C, BARD1 and TP53. The thickness and colour of the ribbons correlate to the correlation of gene expression in the TCGA data set. (H) Highly correlated genes of MCM6 tested by Pearson test in BRCA cohort. (I) The correlation of MCM6 with functional state in BRCA. *p < 0.05, **p < 0.01, ***p < 0.001, versus control.

To investigate the discrepancy expression of MCM6 in tumour versus normal tissues, we analysed the physiological mRNA levels of MCM6 across various normal tissues using the GTEx data set (Figure S1A). As illustrated in Figure 1A, the expression level of the MCM6 gene was found to be significantly upregulated in numerous cancer types, including bladder urothelial carcinoma (BLCA), breast invasive carcinoma (BRCA), cervical squamous cell carcinoma (CESC), cholangiocarcinoma (CHOL), colon adenocarcinoma (COAD), oesophageal carcinoma (ESCA), glioblastoma multiforme (GBM), kidney chromophobe (KICH), kidney renal clear cell carcinoma (KIRC), rectum adenocarcinoma (READ), stomach adenocarcinoma (STAD) and uterine corpus endometrial carcinoma (UCEC). Additionally, data from canSAR.ai indicated that MCM6 expression correlated with the pathological stages of most cancers (Figure S1B). Following this, we utilized canSAR.ai and DISGENET Plus to evaluate the relationship between elevated MCM6 expression and various cancers and diseases. The upregulation of MCM6 was found to have a strong correlation with leukaemia, breast cancer, male‐specific cancers, skin cancer and head and neck cancer, while also demonstrating a significant association with lactose intolerance and some symptom (Figure S1C).

### 2.2 High expression of MCM6 has clinical value in BRCA

To explore the potential role of MCM6 in tumorigenesis and the unclear mechanisms underlying MCM6 dysregulation in the neoplastic process, we utilized the BioGRID online database to construct MCM6‐binding protein–protein interaction (PPI) networks (Figure S2A). Subcluster analysis performed through Metascape indicated that MCM6‐binding proteins were primarily clustered with proteins involved in cell cycle regulation, DNA replication, and DNA damage repair (Figure S2B). Additionally, further Kyoto Encyclopedia of Genes and Genomes and Genome Ontology (KEGG/GO) enrichment analysis, as conducted by Coexpedia and Metascape, revealed that MCM6 and its binding proteins were predominantly associated with DNA replication, DNA damage repair, and cell cycle regulation (Figure 1B, Figure S2C,D).

According to the Coexpedia database and the MeSH (Medical Subject Headings) database, we identified that the medical subject term most strongly correlated with MCM6 was breast cancer (Figure 1C). Consequently, we further investigated the specific association between MCM6 and breast cancer. Our findings demonstrated a significant increase in MCM6 expression in BRCA (Figure 1D). Additionally, data from the Human Protein Altas (HPA) indicated that the staining intensity of MCM6 was markedly elevated in BRCA tissue compared to normal tissues, where it was undetectable (Figure 1E).

Subsequently, we assessed the diagnostic potential of MCM6 in breast cancers through receiver operating characteristic (ROC) analysis. Our results, illustrated in Figure 1F and Figure S3A, indicated that MCM6 may serve as an excellent diagnostic marker across multiple cancers, including breast cancer (AUC >0.5). We further analysed the associations between the overall survival (OS) and progressive free interval (PFI) of patients and MCM6 expression levels using Kaplan–Meier survival analysis. The results demonstrated a significant negative correlation between increased MCM6 expression levels and both OS and PFI in BRCA (Figure 1F and Figure S3B,C). The officially recognized biomarkers for breast cancer include BRCA1, BRCA2, PIK3CA, MKI67, PALB2, ATM, CHEK2, RAD51C, BARD1 and TP53, as reported in the latest publication by The Lancet. To further elucidate the correlation between MCM6 and breast cancer, a relevance analysis revealed that MCM6 exhibits distinct and positive relevance with these biomarkers (Figure 1G).

Next, we conducted a comprehensive bibliometric analysis utilizing the Web of Science (WOS) database. Notably, MCM6 has emerged as a significant trend and focal point in recent years, particularly since 2021. Several countries and research institutions are concentrating on fundamental related to MCM6, while comparatively limited studied address its role in breast cancer (Figure S4A–C).

Subsequently, we employed the Linkedomics database to construct the co‐expression network of MCM6 in BRCA, aiming to elucidate the primary pathways in which MCM6 is involved. The top five positively correlated genes identified are MCM3, MCM5, MCM2, MCM7 and MCM4 (Figure 1H). Furthermore, KEGG analysis revealed enrichment in pathways associated with DNA replication, chromosome segregation, cell cycle checkpoint regulation and DNA damage response (Figure S4D). Analysis of single‐cell sequencing data obtained from cancerSEA, indicates that MCM6 predominantly participates in DNA replication, cell cycle regulation, DNA damage repair and the DNA damage response during the progression of breast cancer (Figure 1I). This evidence suggests that MCM6 is likely to play a critical role in the regulation of DNA replication and the DNA damage response in breast cancer progression.

In conclusion, MCM6 is correlated with an increased risk of breast cancer, indicating that it serves as a putative and vital regulator in breast cancer, especially in regulating its DNA replication and DNA damage response.

### 2.3 Knockdown of MCM6 inhibited proliferation of breast cancer cells

**FIGURE 2:**
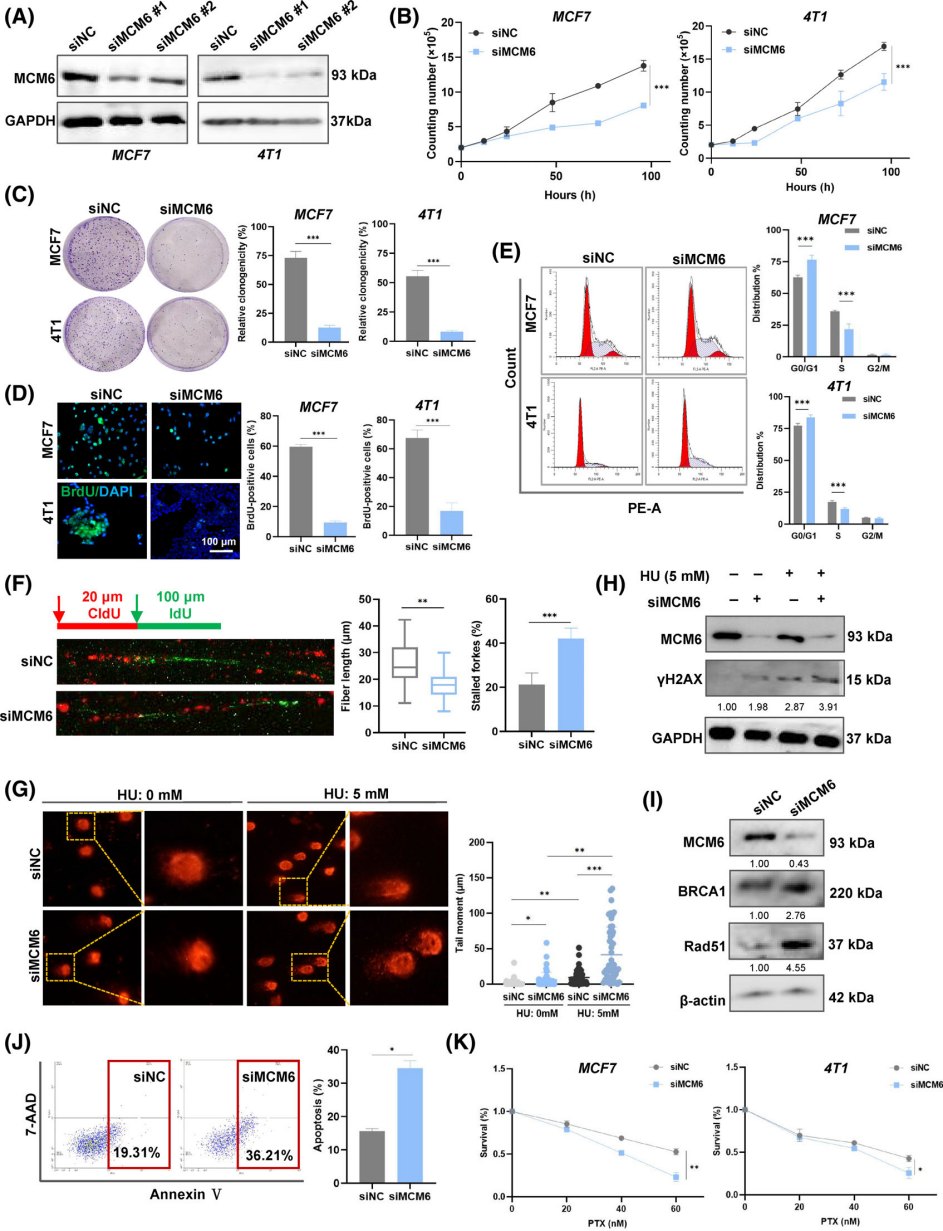
Knockdown of minichromosome maintenance 6 (MCM6) inhibited cells growth and induced cells apoptosis by inducing DNA replication stress. (A) MCF7 and 4 T1 cells transiently transfected with control siRNA (siNC) or MCM6 siRNA (siMCM6#1, siMCM6#2) were subjected to western blot analysis. (B) Cell growth curves were measured by cell proliferation assay. (C–D) Cell proliferation was detected by colony formation (C) and BrdU staining (D). Quantitative analysis of colony numbers is shown in the right panel. For colony formation assays, colonies containing more than 50 cells were counted and plotted. (E) The cell cycle of MCF7 and 4 T1 cells was analysed by flow cytometry. (F) DNA synthesis in MCF7 cells was determined by a DNA fibre assay. Cells were labelled with CldU (20 min) and IdU (40 min). (G) Representative images of neutral comet assay in siNC and siMCM6 cells. Quantification of DNA damage was determined by tail moment using CASP software in the right panel. (H) MCM6‐knockdown MCF7 cells were treated with 5 mM HU, followed by western blotting to confirm γH2AX expression. (I) MCF7 cells were transduced with siRNA against MCM6. The repression of BRCA1 and Rad51 was measured by western blotting. (J) AnnexinV‐PE and 7‐AAD flow‐cytometric analysis of siMCM6 MCF7 cells. (K) Viability of MCF7 and 4T1cells was analysed by MTT 48 h after treatment with different concentrations of paclitaxel. Mean ± SD, *n* = 3. *p < 0.05, **p < 0.01, ***p < 0.001, versus control. BRCA, breast invasive carcinoma.

To confirm the tumour‐promoting role of MCM6, we selected the high expression of the MCM6 breast cancer cell line MCF7 based on data from the Cancer Cell Line Encyclopedia (CCLE) and HPA data sets (Figure S4E,F). We subsequently knocked down MCM6 expression in breast cancer cells MCF7 and 4 T1 cells (The siMCM6#1 was utilized for subsequent research, hereinafter denoted as siMCM6) (Figure 2A), which resulted in suppression of cell growth in vitro (Figure 2B). This observation was corroborated by colony formation assays and BrdU staining, which demonstrated that knockdown of MCM6 inhibited colony formation and reduced the fraction of BrdU‐positive cells (Figure 2C,D). Furthermore, MCM6‐knockdown affected the cell cycle, by decreasing the S‐phase fraction and increasing the G1‐phase fraction (Figure 2E). Our analysis of DNA fibres revealed a substantial reduction in the length of ongoing DNA replication following MCM6 deficiency, indicating that MCM6‐knockdown remarkably obstructed DNA replication and resulted in stalled DNA replication forks (Figure 2F). These findings suggest that aberrant expressed MCM6 regulates breast cancer cell growth and proliferation, while safeguarding DNA replication from DNA replication stress induced by MCM6 deficiency.

### 2.4 Knockdown of MCM6 DNA inducing replication stress caused cell apoptosis

The replication‐coupled DNA damage repair mechanism effectively addresses the challenges posed by DNA replication stress, thereby facilitating the completion of DNA synthesis.^30^ To investigate whether the replication stress induced by knockdown of MCM6 was a consequence of endogenous DNA damage, we assessed DNA damage following depletion of MCM6. As anticipated, MCM6‐knockdown significantly increased DNA damage in MCF7 cells, as evidenced by the length and intensity of the comet tail signal. Furthermore, treatment with hydroxyurea (HU) exacerbated DNA replication stress, a finding corroborated by the DNA comet assay (Figure 2G). This result suggested that MCM6‐knockdown led to the accumulation of DNA damage following HU treatment. Mechanistically, MCM6‐knockdown was associated with elevated levels of γH2AX, a well‐established marker of DNA damage (Figure 2H, Figure S5A). Additionally, the key factors involved in DNA damage repair, BRCA1 and Rad51, remained persistently increased in MCM6‐deficient cells, implying that the absence of MCM6 compromises the efficiency of DNA damage repair (Figure 2I, Figure S5B,C).

Long‐standing DNA damage triggers cell apoptosis, contributing to genomic instability. Flow cytometry analysis, utilizing Annexin V‐PE and 7‐AAD double staining, demonstrated that MCM6‐knockdown induced apoptosis in MCF7 cells, as evidenced by an increase in the populations of 7‐AAD and Annexin V positive cells (Figure 2J). Additionally, TUNEL‐positive cells were identified through green fluorescence staining, revealing a substantial number of TUNEL‐positive cells in MCM6‐depleted breast cancer cells, consistent with the results from Annexin V staining (Figure S5D,E). Moreover, MCM6‐knockdown led to the upregulation of the pro‐apoptosis gene Bax expression while downregulating the anti‐apoptosis gene Bcl‐xl expression (Figure S5F), suggesting that MCM6 serves as a crucial regulatory factor in cell apoptosis.

It is reasonable to speculate that MCM6‐knockdown induced DNA damage triggers cellular apoptosis, enhancing the sensitivity to chemotherapy. Furthermore, resistance to paclitaxel (PTX) contributes to poorer clinical outcomes for patients with breast cancer. MCM6 has been shown to increase the sensitivity of breast cancer cells to PTX in a dose‐dependent manner (Figure 2K). Collectively, these findings suggest that MCM6 plays a crucial role in DNA damage repair and enhances sensitivity to chemotherapy to breast cancer.

### 2.5 MCM6‐Kcr is upregulated upon DNA replication stress

**FIGURE 3:**
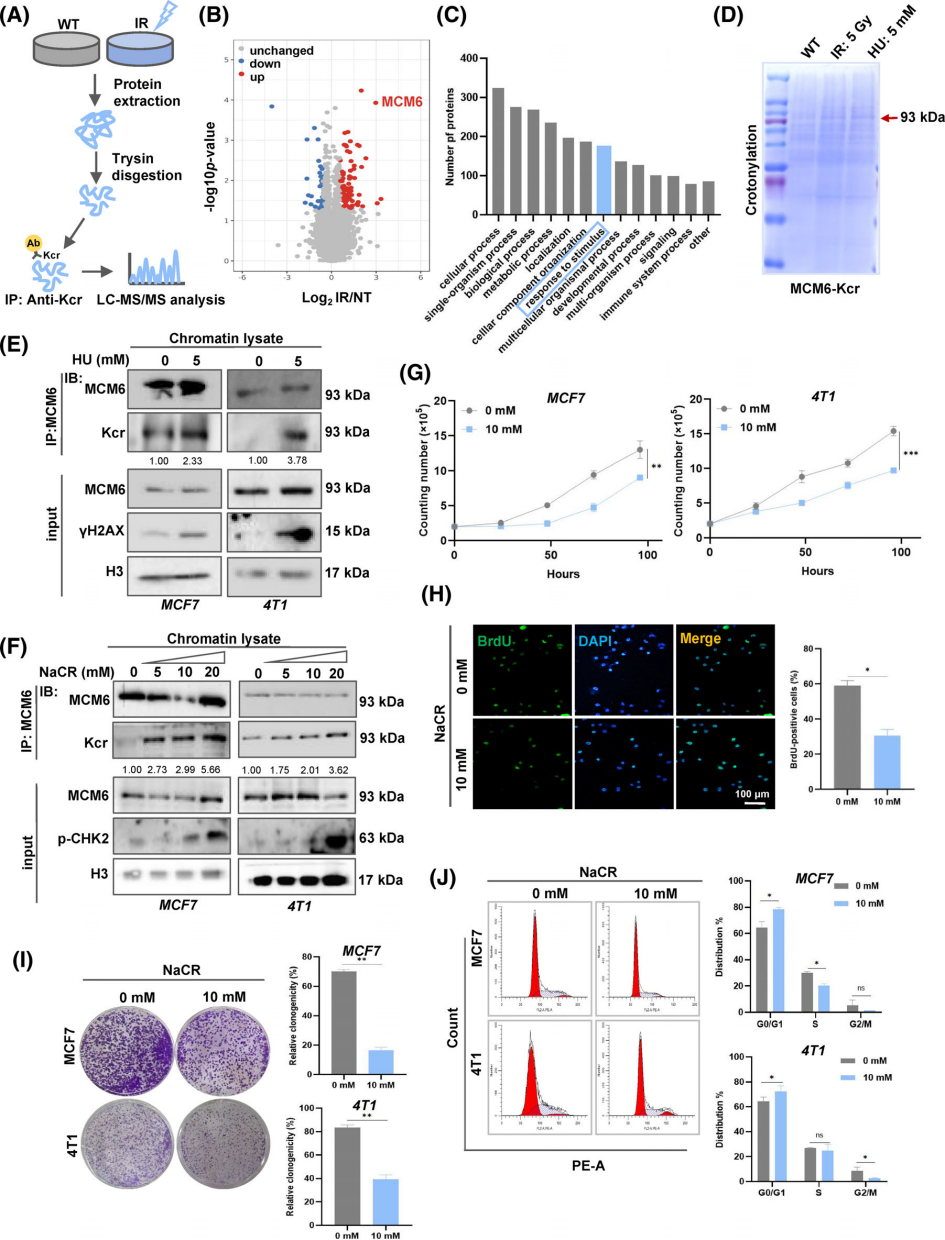
Crotonylation of MCM6 is upregulated upon DNA replication stress. (A) Schematic representation of the experimental workflow for label‐free quantification of Kcr in WT and HU‐treated cells. (B) The differentially expressed proteins analysed by volcano plots as up‐ and down‐regulated proteins. (C) A histogram analysed Kcr‐associated GO pathways enrichment. (D) The expression of different damage‐induced MCM6‐Kcr was verified by coomassie blue staining. (E) Cell chromatin lysates were immunoprecipitated with MCM6 antibody followed by western blotting to confirm γH2AX and Kcr expression. (F) Chromatin lysates of different doses of NaCR were isolated, and co‐immunoprecipitation assay was performed to confirm Kcr and p‐CHK2 expression in MCF7 and 4 T1 cell. (G) Cell growth curves were measured by cell proliferation assay. (H) Cell proliferation was detected by BrdU staining. (I) The cell cycle of MCF7 and 4 T1 cells was analysed by flow cytometry. (J) Colony formation assay to assess cell growth. Quantitative analysis of colony numbers is shown in the right panel. For colony formation assays, colonies containing more than 50 cells were counted and plotted. Mean ± SD, *n* = 3. *p < 0.05, **p < 0.01, ***p < 0.001, versus control.

Since the advancement of modern technology, the characterisation of Kcr has been extensively detailed, however, the functional elucidation of non‐histone modifications remains in its infancy. We employed an integrated approach that combined label‐free quantification, high‐performance liquid chromatography (HPLC) fractionation and high‐resolution LC–MS/MS to investigate Kcr substrates in response to DNA replication stress stimuli (Figure 3A). The results indicated that 85 Kcr sites in 82 proteins were upregulated, while 33 sites in 32 proteins were downregulated in the IR‐treated group compared with the control group (CTRL) (Figure S6A). Notably, crotonylation of MCM6 (MCM6‐Kcr) was extremely upregulated in IR‐treated cells (Figure 3B, Figure S6B). KEGG/GO enrichment analysis revealed that the upregulated Kcr proteins are predominantly involved in various cellular processes, particularly in response to stimuli (Figure 3C, Figure S6C). This finding provides new insight into the role of MCM6‐Kcr, suggesting it may serve as a potential target for breast cancer treatment in regulating DNA damage response.

To further investigate whether MCM6 undergoes crotonylation in response to DNA replication stress, MCF7 cells were subjected to HU and IR, following by immunoprecipitation. The lysates were analysed using coomassie blue staining. The results indicated a significant upregulation of MCM6‐Kcr levels following exposure to IR and HU (Figure 3D). Chromatin‐binding extracts from both control or HU‐treated cells were immunoprecipitated with anti‐MCM6 antibodies and subsequently analysed via immunoblotting with specific antibodies. The findings demonstrated that MCM6‐Kcr levels markedly increased upon HU treatment, suggesting that MCM6‐Kcr significantly heightened the DNA damage (Figure 3E). Furthermore, sodium crotonate (NaCR) was utilized as an exogenous crotonyl donor to promote MCM6‐Kcr, allowing for the assessment of its effects on the viability of breast cancer cells. An increase in NaCR treatment concentration and duration led to a progressive inhibition of the cell cycle due to enhanced MCM6‐Kcr level, as evidenced by the phosphorylation of checkpoint kinase 2 (p‐CHK2), a recognized marker of cell cycle arrest. This implies that MCM6‐Kcr induced DNA replication stress, thereby resulting in cell cycle arrest (Figure 3F, Figure S6D). Additionally, cell proliferation and colony formation assays further confirmed that MCM6‐Kcr suppresses breast cancer cell growth due to cell cycle blockage (Figure 3G–J).

### 2.6 MCM‐K599cr induced DNA replication stress, resulting in cell apoptosis

Based on tandem MS, the putative MCM6‐Kcr site lysine 599 (MCM6‐K599cr) was measured to increase by a factor of 4.007 under HU‐induced DNA replication stress, located at MCM6 AAA^+^ ATPase domain (Figure S7A,B). To demonstrate the evolutionary relationship and conservation of the site, we examined the identified MCM6‐K599 using MEGA 7.0 and WebLogo software, which indicated that MCM6‐K599 was conserved among multiple species (Figure S7C,D). A detailed analysis of the identified MCM6‐K599 site via the PhosphoSitePlus website revealed that MCM6‐K599 could also undergo ubiquitination and acetylation. This suggests that MCM6‐K599 may serve as a hotspot for various PTMs, highlighting the vital functionalities associated with PTMs of MCM6‐K599 (Figure S7E).

**FIGURE 4:**
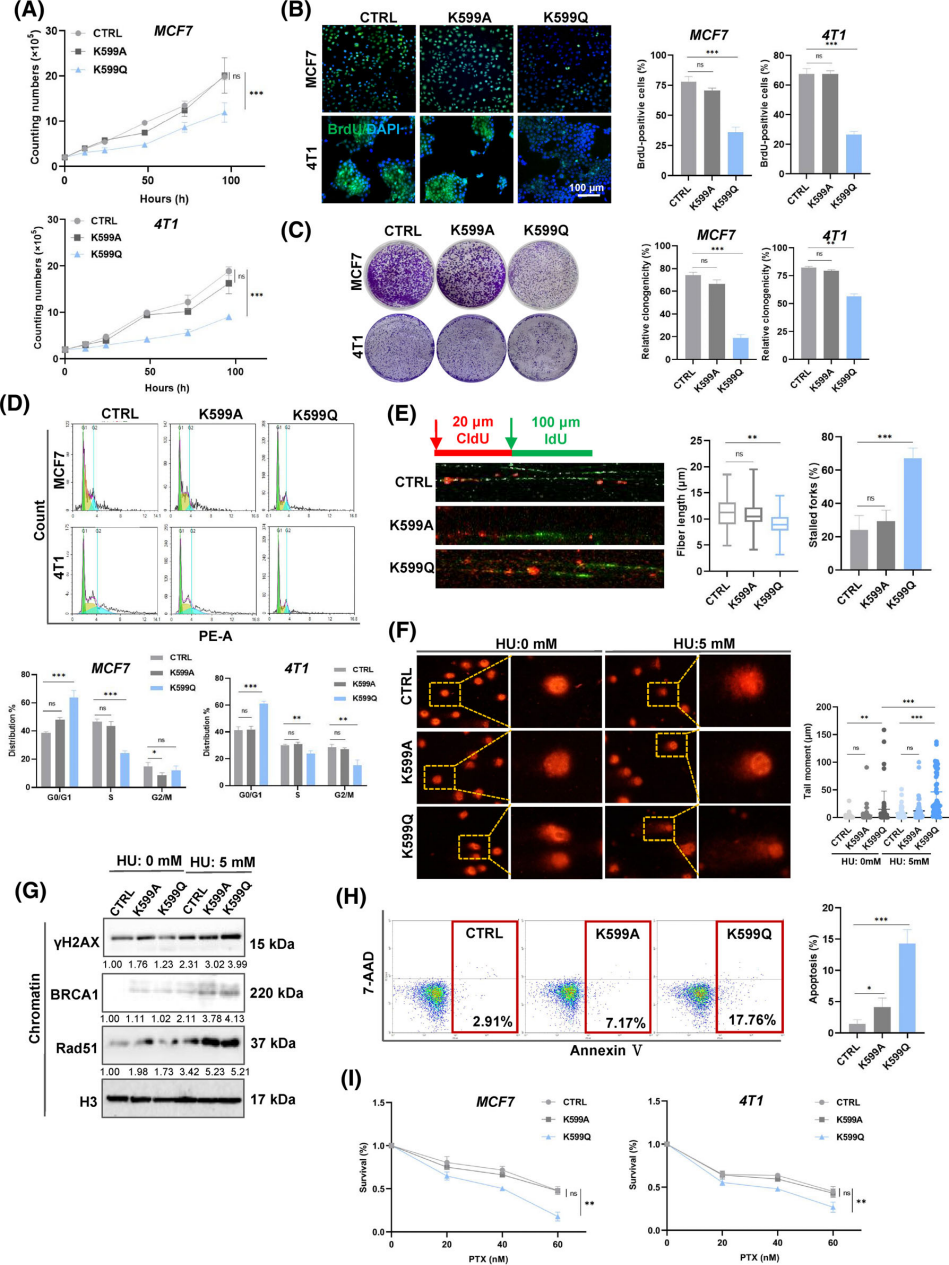
MCM6‐K599cr induced DNA replication stress and caused cells apoptosis. C Cell proliferation was detected by proliferation assay (A), BrdU staining (B) and colony formation (C). Quantitative analysis of colony numbers is shown in the right panel. For colony formation assays, colonies containing more than 50 cells were counted and plotted. (D) The cell cycle of MCF7 and 4 T1 cells was analysed by flow cytometry. (E) DNA synthesis in MCF7 cells was determined by a DNA fibre assay. Cells were labelled with CldU (20 min) and IdU (40 min). (F) Representative images of neutral comet assay in control (CTRL), MCM6‐K599Q (K599Q), and MCM6‐K599A (K599A) cells. Quantification of DNA damage was determined by tail moment using CASP software in the right panel. (G) MCM6‐mutant cells were treated with 5 mM HU, followed by western blotting to confirm γH2AX, BRCA1 and Rad51 expression. (H) AnnexinV‐PE and 7‐AAD flow‐cytometric analysis of all MCF7 cells. (I) Viability of MCF7 cells was analysed by MTT 48 h after treatment with different concentrations of HU and paclitaxel. Mean ± SD, *n* = 3. *p < 0.05, **p < 0.01, ***p < 0.001, versus control. BRCA, breast invasive carcinoma.

To investigate the specific Kcr sites, site‐specific mutagenesis was employed to substitute K599 with glutamine (Q) and alanine (A) to mimic or inhibit crotonylation, respectively. MCF7 and 4 T1 cells transfected with HA‐tagged wild type or point mutant MCM6 were validated by DNA sequencing (Figure S7F). To determine the mechanism underlying the role of MCM6‐Kcr in HU‐induced DNA replication stress and NaCR‐induced cell cycle arrest, we assessed cell growth and proliferation using cell proliferation assays, colony formation assays, and BrdU assays. The results showed that breast cancer cells expressing the MCM6‐K599Q mutant (referred to as K599Q) exhibited reduced cell growth and migration compared to the CTRL and cells expressing the MCM6‐K599A mutant (referred to as CTRL and K599A, respectively) (Figure 4A–C, Figure S8A). Consistently, the K599Q mutant also resulted in cell cycle arrest in the G1 phase, mirroring the phenotype observed with MCM6‐knockdown cells (Figure 4D). Additionally, we employed a DNA fibre assay to investigate whether MCM6‐K599cr induced DNA replication stress. The findings confirmed that the K599Q mutant led to evident DNA replication stress characterised by numerous stalled DNA replication forks. In contrast, both the CTRL and K599A mutant exhibited comparable DNA replication lengths and spreads (Figure 4E).

Our observation of significantly increased comet tail formation in the K599Q mutant indicates DNA damage, accompanied by an extended accumulation of fragmented DNA (Figure 4F). HU further exacerbated DNA damage in the K599Q mutant, as confirmed by γH2AX staining assay. This suggests that MCM6‐Kcr enhances DNA replication stress and aggravates DNA damage (Figure S8B). Further supportive evidence was obtained through western blot analysis of chromatin‐binding proteins, which revealed increased expression of γH2AX, BRCA1, and Rad51 in K599Q mutant cells, particularly following HU‐induced DNA replication stress (Figure 4G). Moreover, BRCA1 and Rad51 were also detected in these cells. Consistent with the γH2AX staining results, the K599Q mutant continued to display numerous foci even after a 12 h repair interval (Figure S8C,D). Collectively, these findings indicate that MCM6‐Kcr induces DNA replication stress and compromises that ability to repair long‐standing DNA damage.

The Annexin V‐PE/7‐AAD flow cytometry assay demonstrated a significant increase in Annexin V‐positive cells (Figure 4H). The TUNEL assay revealed that the K599Q mutant markedly enhanced TUNEL‐positive signals, which were further corroborated by Annexin V staining (Figure S8E,F). In alignment with these results, western blot analysis indicated an increase in Bax expression accompanied by a decrease in Bcl‐xl expression (Figure S8G).

Subsequently, MTT assay results indicated that K599Q mutant cells exhibited a significant reduction in cell viability in response to PTX (Figure 4I). Collectively, these results strongly suggest that MCM6‐K599cr may be implicated in the chemotherapeutic resistance associated with cell apoptosis.

### 2.7 MCM6‐K599cr blocked MCM2‐7 complex assembly, which may be regulated by its ubiquitination

**FIGURE 5:**
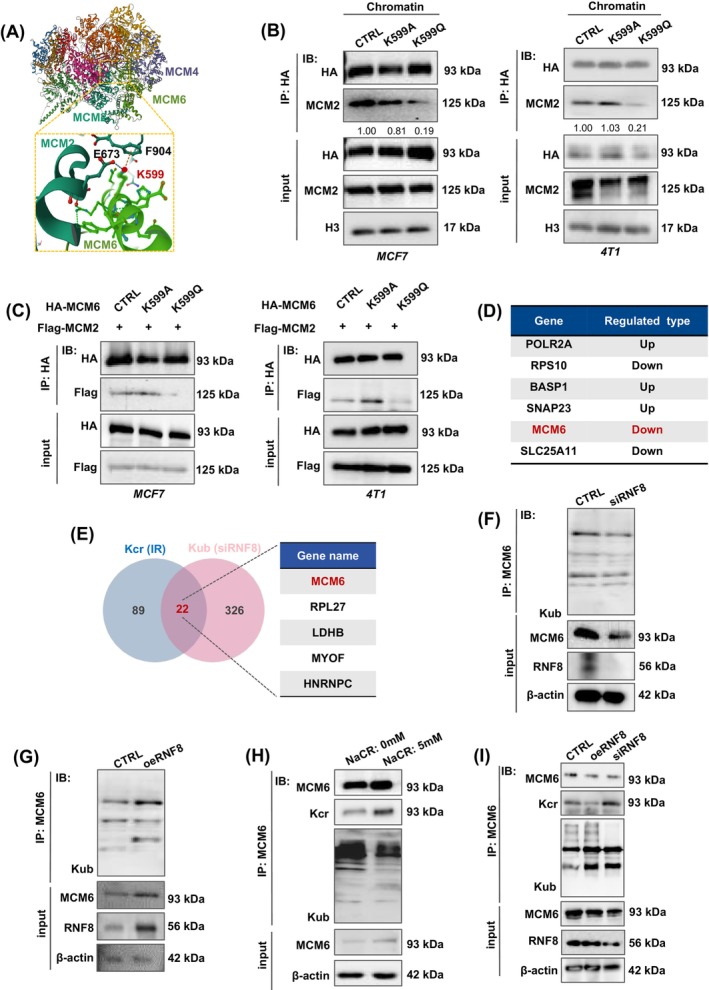
MCM6‐K599cr abolished MCM2‐7 complex formation. (A) Ribbon representation of the MCM2‐7 complex structure. The detailed interaction between MCM6 and MCM2 is shown. (B) Cell chromatin lysates were immunoprecipitated with HA antibody followed by western blotting with Kcr and MCM2 antibodies. (C) In vitro dual transfection system, cells were immunoprecipitated with HA antibody followed by western blotting with specified antibodies. (D) A table summarizing the top 6 proteins identified by this study. (E) A Venn diagram showing overlap between quantifiable Kcr and Kub proteins. (F–G) MCM6 ubiquitination in RNF8‐silenced (siRNF8) MCM7 cells (F) and RNF8‐overexpression (oeRNF8) cells (G). (H) MCF7 cell whole cell lysates were immunoprecipitated with MCM6 antibody followed by western blotting with Kcr and Kub antibodies. (I) MCM6 ubiquitination and crotonylation transformation were detected by immunoprecipitation in siRNF8 and oeRNF8 cells.

MCM6 is a critical component of the MCM2‐7 complex, closely linked with MCM2 and MCM4, respectively. Structural analysis using date from the Protein Data Bank (PDB) revealed that MCM6‐K599 exhibits a strong interaction with MCM2‐E673 and MCM2‐F904 (Figure 5A), implying MCM6‐K599cr may have potential roles to regulate the MCM2‐7 complex assembly. To this end, co‐immunoprecipitation assay indicated that the K599Q mutant disrupts its interaction with MCM2 in chromatin lysates (Figure 5B). Additionally, an exogenous HA‐tag immunoprecipitationassay and reciprocal co‐immunoprecipitation using Flag‐MCM2 confirmed that MCM6‐K599cr inhibited its interaction with MCM2 (Figure 5C). These findings indicate that MCM6‐Kcr is a primary target influencing the formation of the MCM2‐7 complex formation, which subsequently induces replication stress.

As shown in Figure S7E, the MCM6‐K599 site may also be subject to ubiquitination. Our work team has previously investigated RNF8‐mediated protein lysine ubiquitination (Kub) in regulating multiple cellular processes.^31^, ^32^, ^33^ Based on our identical label‐free proteomics analysis of Kub, 351 Kub sites in 272 proteins were downregulated in RNF8‐knockdown cells (siRNF8), including MCM6 (Figure 5D), which are primarily involved in metabolic processes, responses to stimuli, developmental processes and immune system processes (data not show). The comparison of the Kcr and Kub results revealed 22 overlapping proteins. Notably, MCM6 could also undergo ubiquination in an RNF8‐dependent manner (Figure 5E).

To investigate the mechanism of RNF8‐dependent MCM6‐Kub in MCF7 cells, we first generated RNF8‐knockdown and RNF8‐overexpressing mutants (referred to as siRNF8 and oeRNF8, respectively), which were then transfected into MCF7 cells. The co‐immunoprecipitation assay results showed that depletion of RNF8 remarkably inhibited MCM6‐Kub, a process that be rescued by overexpressing of RNF8 (Figure 5F,G). Given that both MCM6‐Kcr and MCM6‐Kub are present concurrently in the cells, we next sought to determine whether the dynamic transformation between MCM6‐Kcr and MCM6‐Kub is regulated by RNF8. Co‐immunoprecipitation assays demonstrated an increase in the MCM6‐Kcr level. Conversely, MCM6‐Kub decreased following treatment with 5 mM NaCR (Figure 5H). This observation aligns with the finding that RNF8 deficiency leads to reduced MCM6‐Kub levels alongside elevated MCM6‐Kcr levels (Figure 5I). These results further support the idea that MCM6‐Kcr and MCM6‐Kub may influence one another through RNF8, suggesting that RNF8 could represent a novel target for therapeutic intervention in breast cancer.

### 2.8 The crotonylation eraser SIRT7 decreased MCM6‐Kcr during DNA replication stress

**FIGURE 6:**
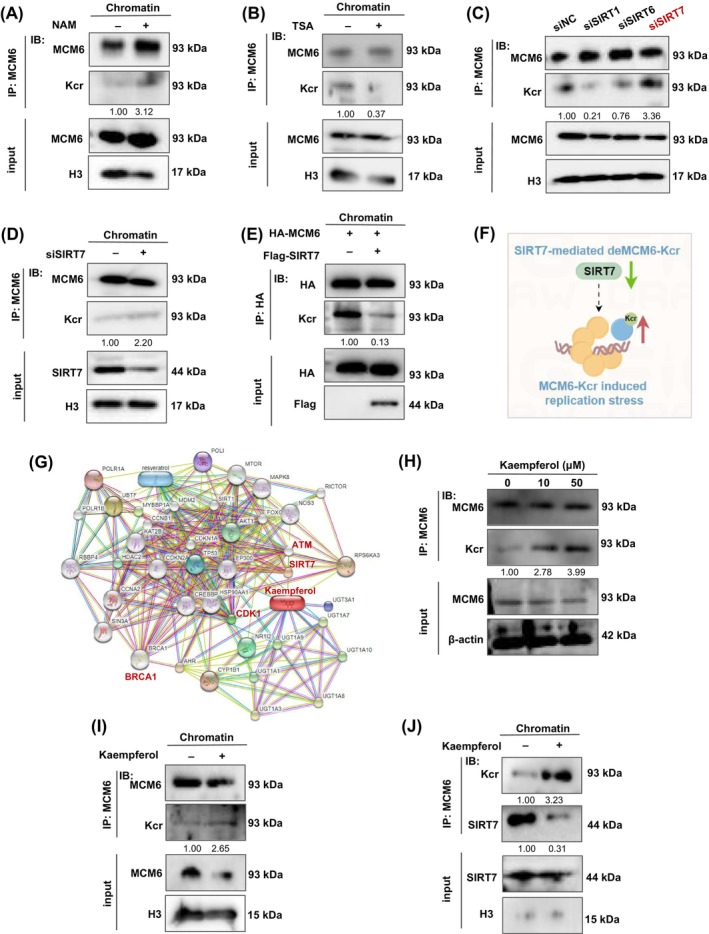
The crotonylation eraser SIRT7 decreased MCM6‐Kcr levels and regulated by kaempferol. (B) MCF7 cells were incubated with or without Nicotinamide (NAM, 10 mM for 24 h, (A) or Trichostatin A (TSA, 1 μM for 24 h, (B). After treatment, chromatin proteins were extracted and subjected to western blot analysis using the indicated antibodies. (C) Whole cell lysates were prepared from MCF7 cells transfected with the indicated siRNAs and immunoblotted with the indicated antibodies. (D) Chromatin‐binding proteins were prepared transfected with siSIRT7 and immunoblotted with the Kcr. (E) In vitro dual transfection system, cells were immunoprecipitated with HA antibody followed by western blotting with specified antibodies. (F) Schema chart. SIRT7‐mediated decrotonylation of MCM6 resulted in DNA replication stress. (G) Correlation between SIRT7 and kaempferol via STITCH database. (H) MCF7 cells treated different concentration of kaempferol for 24 h; then analysed by immunoprecipitation using the indicated antibodies. (I) Chromatin fractions were extracted from cells and analysed by immunoprecipitation using MCM6 to immunblot Kcr. (J) Chromatin fractions were extracted from cells and analysed by immunoprecipitation using MCM6 to immunblot SIRT7.

Previous studies have demonstrated that certain histone deacetylases exhibit de‐crotonylation activity.^34^ To clarify the decrotonylase of MCM6, we treated cells with the specific HDAC family inhibitor Trichostatin A (TSA) and the SIRT family inhibitor Nicotinamide (NAM) to identify the decrotonylase responsible for MCM6. Co‐immunoprecipitation experiments involving chromatin‐binding proteins revealed that treatment with 10 mM NAM resulted in an increased MCM6‐Kcr level, whereas treatment with 1 μM TSA did not (Figure 6A,B). This suggests that the SIRT family may function as a potential decrotonylase for MCM6.

Currently, seven mammalian SIRT members named SIRT1 to SIRT7 have been identified. As far as we know, SIRT1, SIRT6 and SIRT7 are mostly located in the chromatin, whereas SIRT3‐5 localizes primarily to mitochondria and SIRT2 is mostly found in the cytoplasm. Meanwhile, some reports also indicated that SIRT1,^35^ SIRT6^36^, ^37^ and SIRT7^38^, ^39^, ^40^ were considered as the central regulator of DNA damage repair and were usually caused by the overproduction of reactive oxygen species (ROS).^41^ Thus, we designed siRNA to target knockdown SIRT1, SIRT6 and SIRT7. Knockdown of SIRT7 but not SIRT1 or SIRT6 significantly rescued the level of MCM6‐Kcr (Figure 6C). MCM6‐Kcr levels were significantly elevated in the chromatin segment of cells with SIRT7 depletion (Figure 6D). To validate this observation, an in vitro decrotonylase assay was conducted using a dual transfection model, which further corroborated SIRT7 inhibiting MCM6‐Kcr (Figure 6E). Consequently, we concluded that SIRT7 functions as a specific decrotonylase for MCM6, thereby modulating MCM6‐Kcr and inducing replication stress (Figure 6F).

### 2.9 Kaempferol targeted SIRT7 to modulate MCM6 crotonylation

Several natural products have been reported to reverse chemoresistance by inhibiting proliferation, regulating DDR‐related proteins and inducing apoptosis in cancer cells, such as flavonoids compounds.^42^, ^43^ Natural products have unique advantages with low toxicity and multi‐target features, making them widely used against chemoresistance. Therefore, we aimed to identify a small molecule compound targeting SIRT7 to modulate the MCM6‐Kcr, thereby reversing chemotherapeutic resistance. Utilizing the STITCH, SymMap V2 and CTD database, we identified kaempferol as a potential ingredient targeting SIRT7 (Figure 6G, Figure S9A). Molecular docking analysis using CB‐dock 2.0 revealed that kaempferol binds to SIRT7 through two binding pockets with a strong binding affinity, as indicated by an absolute vina score less than −7.0 (vina score = −8.4; vina score = −7.6) (Figure S9B).

As illustrated in Figure 6H, MCM6‐Kcr exhibited a clear dose‐dependent response to kaempferol, while no significant difference in MCM6 level was observed. A similar result was obtained through chromatin protein detection (Figure 6I). As excepted, kaempferol upregulated MCM6‐Kcr by inhibiting SIRT7 expression (Figure 6J).

### 2.10 Kaempferol suppressed proliferation and induced DNA replication stress in breast cancer cells

Kaempferol, a flavonoid compounds, has been shown to inhibit the proliferation of oral cancer cells. It also increased the expression of γH2AX and caspase‐3, leading to DNA damage and inducing cell apoptosis.^44^ However, the precise mechanism by which kaempferol exerts therapeutic effects in breast cancer, potentially through the modulation of SIRT7‐mediated MCM6‐Kcr, remains to be fully elucidated.

Based on BATMAN database and DO analysis (Figure S10A,B), a total of 52 genes were recognized as candidate targets, while 64 disease targets were found in CTD database and 59 disease targets in OMIM database, all of which are closely related to breast cancer. Pathway enrichment analysis from KEGG/GO enrichment indicated that kaempferol is primarily involved in pathways in cancer, response to stimuli, cellular process and cell growth (Figure S10C,D). These findings suggest that kaempferol may regulate DNA replication stress, hereby modulating the progression of breast cancer.

**FIGURE 7:**
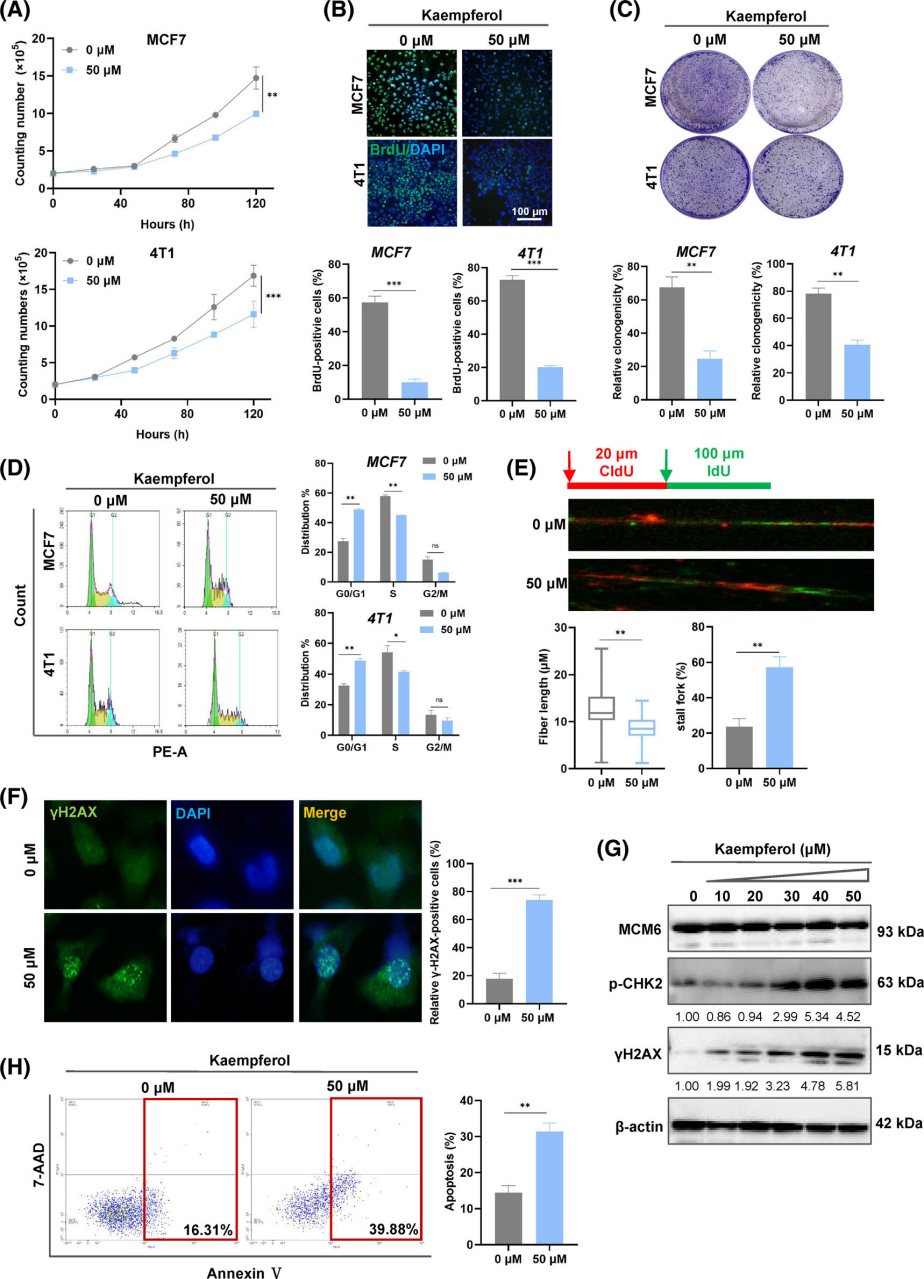
Kaempferol suppressed proliferation and induced DNA replication stress in breast cancer cells. (A–C) Cell proliferation was detected by proliferation assay (A), colony formation (B) and BrdU staining (C). Quantitative analysis of colony numbers is shown in the right panel. For colony formation assays, colonies containing more than 50 cells were counted and plotted. (D) The cell cycle of MCF7 and 4 T1 cells was analysed by flow cytometry. (E) DNA synthesis in MCF7 cells was determined by a DNA fibre assay. Cells were labelled with CldU (20 min) and IdU (40 min). (F) MCF7 cells were treated with 50 μM kaempferol, following processed for immunofluorescence using γH2AX. (G) MCF7 cells were treated with different concentration of kaempferol, followed by western blotting to confirm γH2AX and p‐CHK2 expression. (H) Annexin V‐PE and 7‐AAD flow‐cytometric analysis of all MCF7 cells. Mean ± SD, *n* = 3. *p < 0.05, **p < 0.01, ***p < 0.001, versus control.

Further validation of the effect of kaempferol was conducted through proliferation and cell cycle assays. The results showed that kaempferol inhibits cell growth and blocks cell cycle (Figure 7A–D). We subsequently assessed DNA replication stress and DNA damage. As illustrated in Figure 7E,F, kaempferol suppressed DNA synthesis and induced a significant accumulation of stalled DNA forks, leading to replication‐stress‐induced DNA damage in a dose‐dependent manner (Figure 7G). We also investigated whether kaempferol induced cell apoptosis similar to MCM6‐Kcr. As anticipated, kaempferol resulted in cell apoptosis (Figure 7H).

### 2.11 Kaempferol inhibited tumour growth via enhancing PTX sensitivity

**FIGURE 8:**
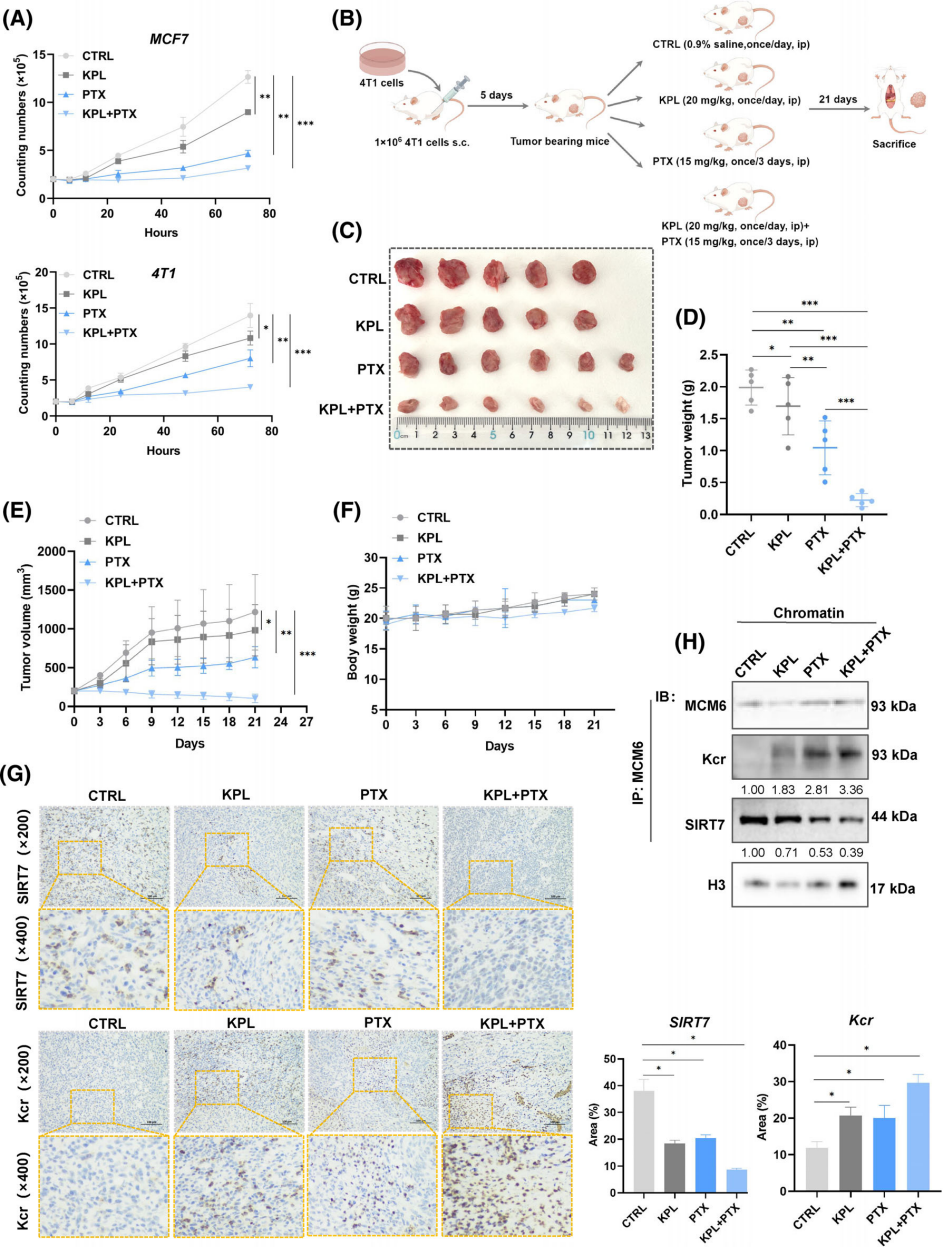
Kaempferol inhibited tumour growth via enhancing paclitaxel (PTX) sensitivity. (A) Cell proliferation was detected by proliferation assay after treatment with 50 μM kaempferol or 10 nM paclitaxel alone, 50 μM paclitaxel and 10 nM kaempferol combination. (B) Schematic diagram of 4 T1‐bearing mice modelling and in vivo experimental procedure. (C) Representative images of the tumour tissues in each group. (D–E) Representative images of the tumour weight (D) and volume (E) in each group. (F) Body weight changes in each group. (G) Immunohistochemistry images of SIRT7 and Kcr expression in each group. (H) Extraction the proteins from each group tissues, then analysed by immunoprecipitation using the indicated antibodies. Mean ± SD, *n* = 3. *p < 0.05, **p < 0.01, ***p < 0.001, versus control.

Under identical experimental conditions, strong enhanced antiproliferative activity of kaempferol (KPL) on PTX regulation of cell proliferation was demonstrated in MCF7 and 4 T1 cells (Figure 8A). Encouraged by these results, we subsequently assessed the antitumor effects of paclitaxel‐kaempferol (PTX‐KPL) treatment in vivo. 4 T1 cells were subcutaneously injected into the flank of BALB/c mice to establish the tumour model. The tumour‐bearing mice were randomly divided into four groups and subjected to the injection schedule outline in Figure 8B. Tumour measurements were taken after 21 days post‐injection (Figure 8C). Compared to the CTRL, both tumour weight and volume exhibited a gradually decrease (*p* < 0.05) following treatment with either PTX or KPL alone, while the PTX‐KPL combination therapy demonstrated significant anti‐tumour effects (*p* < 0.01) (Figure 8D,E).

Additionally, we evaluated the potential toxicity of the different formulations. Body weight did not significantly differ among the groups, indicating that all treatments were devoid of evident toxicity (Figure 8F). Furthermore, the IHC and co‐immunoprecipitation assay corroborated previous findings, showing that kaempferol enhanced potency of PTX by reducing SIRT7 expression, thereby inducing MCM6‐Kcr‐mediated DNA replication stress (Figure 8G,H).

## 3 DISCUSSION

Breast cancer represents the most prevalent malignancy and the leading cause of cancer‐related mortality among women. Chemotherapeutic resistance poses a severe challenge in the treatment of breast cancer. Numerous studies have indicated that dysregulation of the MCM6 complex is frequently observed in various tumours.^45^ MCM6 acts as a universal biomarker for tumour growth, owing to its critical roles in DNA replication and cell cycle regulation.^46^, ^47^ However, a comprehensive analysis of MCM6 in the context of breast cancer has yet to be conducted in the existing literature.

Although a negative aspect of replication stress is its prominent role in tumorigenesis, a positive aspect is that it provides a potential target for cancer therapy. Previous studies have demonstrated that overactive DNA damage repair genes in tumour stem cells may significantly contribute to the limitation of replication stress in breast cancer stem cells.^48^ Azzoni et al.,^49^ confirmed that inhibiting RAD51 expression can effectively intensify DNA replication stress in breast stem cells, thereby enhancing their sensitivity to cisplatin. Venugopal et al.,^50^ discovered that the DNMA3T^R882^ mutation not only exacerbates the accumulation of DNA replication stress but also increases the sensitivity of acute myeloid leukaemia (AML) cells to the chemotherapeutic agent HU. In this study, we observed that knockdown of MCM6 led to the accumulation of endogenous DNA damage due to DNA replication stress, resulting in cell apoptosis and heightened sensitivity of breast cancer cells to the chemotherapeutic drugs. The aforementioned studies collectively suggest that targeted therapies aimed at addressing DNA replication stress and damage repair offer significant promise for cancer treatment.

Although Kcr has been widely studied, the detailed biological functions of Kcr modification, especially non‐histone Kcr modification, remain largely unknown. Xu et al.,^51^ identified 1024 protein modifications with altered crotonylation levels in the lung adenocarcinoma cell line H1200, marking the first demonstration that non‐histone proteins involved in various signalling pathways can be modified by crotonylation. Zheng et al.,^29^ conducted crotonylation proteomics in pancreatic cancer cells and performed KEGG/GO enrichment analysis on these Kcr proteins, which are involved in glycolysis and the TCA cycle, including 1DH1 and MTHFD1. The crotonylation of these proteins may play a significant role in the metabolism and progression of tumour cells. Chen et al.,^52^ identified 1109 Kcr modification sites on 347 proteins in maintenance haemodialysis patients, revealing that these proteins were closely associated with complications arising during haemodialysis. While some studies have indicated that proteins involved in cell replication stress and DNA damage repair also undergo Kcr modification, the mechanisms and precise roles of Kcr protein in these processes remain unclear. In our study, we identified increased Kcr proteins are mainly involved in stimulus response, in particular, MCM6‐Kcr was upregulated under DNA replication stress. Further assays confirmed that MCM6 was crotonylated in breast cancer cells, with significantly elevated levels of MCM6‐Kcr observed following HU treatment and triggering cellular replication stress, leading to DNA damage, cell cycle arrest and suppression of breast cancer cell proliferation.

The MCM6‐K599 site is highly conserved across multiple species and is subject to various PTMs types, including phosphorylation, ubiquitination and acetylation. This indicates that the PTMs at this site are critical for regulating MCM6 function. Currently, there are no studies documenting the Kcr and its functional implications for MCM6. MCM6‐K599cr induced severe DNA replication stress, accumulating numerous stalled DNA replication forks. Given our observations, the K599Q mutant also induced cell apoptosis and exhibited significantly increased sensitivity to chemotherapy drug.

Our work team previously focused on the RNF8‐mediated protein Kub. To our surprise, we found that MCM6 could also be ubiquitinated through the preceding MS‐based proteomics analysis of ubiquitination, which attracted our attention to how two distinct modifications regulate the function of MCM6. The absence of RNF8 led to a reduction in the levels of MCM6‐Kub, while SIRT7‐mediated MCM‐Kcr dynamically competes with RNF8‐mediated MCM6‐Kub. We postulate that under conditions of DNA replication stress and DNA damage, the levels of MCM6‐Kcr increase, thereby inhibiting the formation of the MCM2‐7 complex and obstructing the unwinding of DNA double strands to prevent subsequent errors in DNA replication. Conversely, during normal DNA replication, it is possible that MCM6‐Kub inhibits MCM6‐Kcr to prevent DNA replication stress‐induced endogenous DNA damage and to maintain genome stability. However, in this study, we found that the absence of RNF8 could also affect the expression of MCM6. RNF8 plays a critically important role in transducing DNA damage signals, interacts with UBCH8 and UBC13, and catalyses both K48‐ and K63‐linked ubiquitin chains.^53^ However, this study did not explore the precise regulatory on RNF8‐mediated MCM6, this aspect warrants further investigation as a potential research direction.

Kaempferol displays several pharmacological properties, among them anti‐microbial, anti‐inflammatory, anti‐oxidant, anti‐tumour, cardioprotective, neuroprotective and anti‐diabetic activities, and is being applied in cancer chemotherapy.^54^ Network pharmacology and further experiments indicated that kaempferol could inhibit breast cancer cells proliferation and induce DNA replication stress as MCM6‐knockdown and MCM6‐Kcr did. Kaempferol upregulated MCM6‐Kcr by inhibiting SIRT7, thereby inducing DNA replication stress and cell apoptosis in breast cancer cells. Finally, the in vivo model also verified our observations that kaempferol has limited antitumor ability while effectively enhancing PTX efficacy by inducing MCM6‐Kcr induced DNA replication stress. However, in vivo studies and clinical trials using more approaches are scarce so far, thus stressing the need for more in‐depth experiments to explore more precise functions of kaempferol.

Additionally, we are aware of some limitations of the present study, including the fact that only limited cell lines were used to investigate MCM6 functions in breast cancers. Breast cancer can be categorised into three main types and five subtypes characterised by alterations in the expression of specific genes and the presence or absence of surface receptors, which are classified into HER2 positive (HER2^+^), luminal types and triple‐negative breast cancer (TNBC).^55^ However, we did not mainly focus on the specific cancer type, which still needs more research for further investigation. Moreover, our study indicated that RNF8‐mediated MCM6‐Kub regulated its Kcr level, whereas the detailed modification sites and potential mechanisms have yet to be characterised. Since K599 is one hotspot for various PTMs, we found that whether the K599 site is the Kub site competing with its Kcr is still unknown.

In summary, our study is a novel systematic analysis of MCM6 and its Kcr in regulating DNA replication stress and the DNA damage response for breast cancer treatment. Further investigation of the functions of MCM6‐Kcr in diverse cellular pathways will deepen our understanding of the complex PTM code and provide clues for future drug development to combat diseases, including cancer.

## 4 METHODS

### 4.1 Gene expression level analysis

The mRNA levels of MCM6 in a variety of cancers were analysed by Tumour Immune Estimation Resource, version 2 (TIMER2.0, TIMER2.0 (cistrome.org)).^56^ Differential expression levels of MCM6 were observed between the cancerous region and normal tissue for various types of tumours. The range was set as follows:log2‐fold change (log2 FC) ≥ 1 or ≤ −1 and *p* value ≤0.05. Gene Expression Profiling Interactive Analysis, version 2 (GEPIA2, GEPIA 2 (cancer‐pku.cn))^57^ was employed to analyse the MCM6 mRNA levels in certain tumours with no corresponding normal tissues. Additionally, violin plots were applied to reveal the relationship between MCM6 and pathological stages of cancers by using the “Stage Plot” and “PR status” of GEPIA. In addition, canSAR.ai (canSAR.ai | the Cancer Drug Discovery Platform)^58^ and DisGeNET (DisGeNET—a database of gene‐disease associations)^59^ were utilized to analyse the correlations of MCM6 with cancers and diseases.

### 4.2 Survival prognosis analysis and gene enrichment analysis

The OS and DFS of all tumour patients in the GEO cohorts were analysed using the Kaplan–Meier plotter (Kaplan–Meierplotter[Breast](kmplot.com)) and PrognoScan tools (PrognoScan: A new database for meta‐analysis of the prognostic value of genes. (kyutech.ac.jp)). Furthermore, we visualized the protein–protein interaction network using Coexpedia software (Coexpedia)^60^ and clarified the potential pathways of the top 100 MCM6‐binding proteins using Metascape (Metascape)^61^ and GPS Heml2.0 (HemI 2.0—Heatmap Illustrator(biocuckoo.cn)).^62^

### 4.3 Cell lines, culture method, and reagents

The human embryonic kidney cell line HEK293 and the human breast cancer cell lines MCF7 and MDAMB231 were purchased from ATCC (Manassas, VA). All cell lines were cultured in Dulbecco's modified Eagle's medium (DMEM, Servicebio) containing 10% fetal bovine serum (FBS, HyClone) supplemented with 5 μL penicillin/streptomycin (Servicebio) in a 5% CO2 atmosphere at 37°C.

Sodium crotonate (NaCR, Absin, abs42074236), hycamptothecin (HCPT, Solarbio, IH2120), hydroxycarbamide (HU, Solarbio, H8420) and paclitaxel (PTX, Solarbio, YZ‐100382) were dissolved in DMSO and used at the specified concentrations and times as indicated. The following primary antibodies were used for western blotting: MCM6 polyclonal antibody (Proteintech, 13347‐2‐AP), Anti‐Crotonyllysine Mouse mAb (panKcr, ptm‐biolab, PTM‐502), RAD51 Polyclonal antibody (proteintech, 14961‐1‐AP), Anti‐BRCA1 (Abcam, ab238983), Anti‐53BP1 (Abcam, ab175933), anti‐gamma H2A.X (phospho S139) (γH2AX, Abcam, ab81299), Anti‐Chk2 (phospho T383) (p‐CHK2, Abcam, ab59408), Beta Actin recombinant antibody (β‐actin, Proteintech, 81115‐1‐RR), Histone‐H3 Polyclonal antibody (proteintech, 17168‐1‐AP) and GAPDH Monoclonal antibody (Proteintech, 60004‐1‐lg). The following second antibodies were used for western blotting: HRP‐conjugated Affinipure Goat Anti‐Mouse IgG (H + L) (Proteintech, SA00001‐1) and HRP‐conjugated Affinipure Goat Anti‐Rabbit IgG (H + L) (Proteintech, SA00001‐2).

### 4.4 Transfection with siRNA and plasmid DNA

The complementary DNA (cDNA) for control MCM6 was amplified by polymerase chain reaction and ligated into pcDNA plasmid containing HA‐tag. For construction of the HA‐MCM6‐K599A and HA‐MCM6‐599Q plasmids, site‐directed mutagenesis (Sino Biological) was applied and verified by sequencing. For generation of stable MCF7 RNF8 knockout cells, sgRNF8 were subcloned and inserted into the lentiCRISPRv2 vector. For transient transfection with plasmid DNA, MCF7 (K599A and K599Q) and MCF7 (siRNF8 and oeRNF8) cells were seeded in 6‐well plates at a confluency of 70%–80% and transfected with plasmid DNA using Lipofectamine 2000 (Invitrogen) in Opti‐MEM medium according to the manufacturer's protocol. After 48 h, MCM6 and RNF8 expression was evaluated by western blotting, and cells were used for downstream applications. For transfection with siRNAs, multiple predesigned siRNAs per target gene as well as scrambled control siRNA were obtained from Genepharma. MCF7 (siMCM6) cells were transfected at 70%–80% confluency in six‐well plates with 50 nM of siRNA using GP‐transfect‐Mate (Genepharma) in Opti‐MEM medium following the manufacture's protocol. Downregulation of target genes was analysed by western blotting and immunofluorescence microscopy 48 h after transfection.

### 4.5 Extraction of chromatin‐bound proteins by biochemical fractionation

Cell fractionation was performed as described in the Bioprotocol.^63^ Briefly, cells were harvested, washed with PBS and resuspended in E1 buffer (50 mM HEPES‐KOH pH 7.5, 140 mM NaCl, 1 mM EDTA pH 8.0, 10% glycerol, 0.5%NP‐40, 0.25% Trixon X‐100, 1 mM DTT, protease inhibitor cocktail) on ice for 10 min, and soluble proteins were separated by centrifuge at 1100 g. The nucleocyte proteins were extracted with E2 buffer (10 mM Tris–HCl pH 8.0, 200 mM NaCl, 1 mM EDTA, 0.5 mM EGTA, protease inhibitor cocktail) with the same procedure as before. The chromatin‐bound pellet was isolated by ice E3 buffer (50 mM Tris–HCl pH 7.5, 20 mM NaCl, 1 mM MgCl_2_,1% NP‐40, protease inhibitor cocktail, 1:1000 bezonase) followed by sonication and centrifugation at 16000 × g.

### 4.6 Immunoprecipitation (IP) and immunoblotting (IB)

Cells were washed with ice‐cold PBS, lysed in RIPA buffer (50 mM Tris–HCl pH 7.4, 0.5% Na‐deoxycholate, 0.1% SDS, 150 mM NaCl, 2 mM EDTA and 50 mM NaF) supplemented with protease inhibitor cocktail and MG132 (MCE, HY‐13259), scraped and incubated in cold tubes for 30 min on ice. Cell lysates were centrifuged at 12000 rpm at 4°C for 20 min, and the supernatant was used to determine the protein concentration by spectrophotometer (DeNovix, DS‐11). SDS sample buffer was added to the lysates, and standard immunoblotting procedures were followed. For IP, cells were lysed and quantified for protein concentration. The primary antibody was added and incubated in a 4°C rotation rack overnight. Protein G beads were then added for an additional 3 h at 4°C with rotation. The resin was then centrifuged for 4 min at 550 × g, washed three times with lysis buffer and analysed by immunoblotting.

### 4.7 Immunofluorescence microscopy

Immunofluorescence analyses were performed as previously described.^64^ Briefly, the cells were cultured on coverslips at a concentration of 2 × 10^5^ cells/well in a 6‐well plate, and the cells were washed with PBS. Following the removal of PBS, the cells were fixed in 1 mL of 100% ice‐cold methanol at −20°C for 7 min and then blocked with 2% BSA solution in PBS containing 0.1% Tween‐20 at 4°C overnight. The cells were then incubated with the appropriate antibodies at 4°C overnight. After washing three times with blocking buffer, the cells were incubated with appropriate secondary antibodies for 1 h at 37°C in the dark and then incubated in DAPI solution for 2 min. Cells were subjected to immunofluorescence analyses using a fluorescence microscope.

### 4.8 DNA fibre assay

DNA fibre analyses were performed as previously described.^65^ Briefly, cells were transfected with target siRNA or plasmid, pulse‐labelled with CldU, and then pulse‐labelled with IdU in fresh medium. After trypsinization, the cells were washed and resuspended in PBS. Then, 2 μL of cell suspension was placed on a glass slide, which was angled to allow DNA to spread, and lysis buffer. DNA was denatured with HCl and blocked with 5% bovine serum albumin (BSA) in PBS for 1 h after washing. Slides were stained with antibodies and sealed with coverslips. Slides were imaged using a microscope and analysed using ImageJ software. At least 200 replication forks were analysed per experimental condition, while the analysis shows the mean of three independent experiments.

### 4.9 Neutral comet assay

Neutral comet assays were carried out as previously described.^66^ Briefly, 2 × 10^5^ cells were suspended in PBS, mixed with 1% low‐melting‐point agarose, pipetted onto a slide precoated with 1% agarose, and then covered with 0.5% low‐melting‐point agarose. The cells were lysed with lysis solution (2.5 M NaCl, 0.1 M EDTA, 10 mM Tris, 10% DMSO, 1% Triton‐X100) at 4°C for 2 h. Excess ions were washed off with double‐distilled water, the slides were placed in a horizontal electrophoresis tank containing electrophoresis solution (1 mM Na_2_EDTA, 300 mM NaOH) to soak for 20 min, and electrophoresis was run for 40 min. Following electrophoresis, the slides were rinsed with neutralization buffer (0.4 M Tris, pH 7.5) for 15 min. The slides were the washed in distilled water for 5 min and stained with propidium iodide (PI, 5 μg/mL) for 20 min, and then excess PI was washed off with distilled water. The slides were observed under 590 nm excitation light using a fluorescence microscope. At least 100 comet images from each slide were analysed using comet image analysis software.

### 4.10 Apoptosis assay

Apoptosis was detected by a TUNEL (terminal deoxynucleotidyl transferase nick‐end labeling) fluorescein kit (ZIKER, ZK‐PL4019) and confirmed with the Annexin V‐PE/7‐AAD detection kit (Sino Biological, APK10448‐P) using a fluorescence microscope and flow cytometer.

### 4.11 Tumorigenesis and treatment

All mice were kept for 1 week for environment adaptation before starting the injections. After adaptation, Baclb/c mice were inoculated into their left fat pad with 4 T1 cells (1 × 10^6^ in 100 μL PBS). Regular checks were performed to assess tumour growth until the palpable tumour was detected. After 5 days of injections, all the mice were randomly divided into 4 groups, tumour size were measured every three days. Then, 5 mice in the CTRL were injected with 0.9% normal saline every day; 5 mice in the kaempferol single group (KPL) were injected with 20 mg/kg kaempferol every day; 6 mice in the paclitaxel single group (PTX) were injected with 15 mg/kg every three days; 6 mice in kaempferol and PTX combination group (KPL + PTX). In the third week, mice were killed after anaesthesia, and then the tumour tissues were collected for further analysis.

### 4.12 Statistical analysis

Quantified results are presented as the mean ± SEM of at least three duplicates. Comparisons between groups were performed with an unpaired Student's t‐test. The results were considered statistically significant only if the *p* value was less than 0.05, and the significance level is indicated as *p < 0.05, **p < 0.01, ***p < 0.001.

## AUTHOR CONTRIBUTIONS

HY.S and Z.G were responsible for the manuscript; HY.S designed the project in collaboration with R.S and DG.W; HY.S and Z.G performed the cell experiments; K.X and XW.L contributed to the bioinformatics analysis; XG.Y and K.X contributed reagents; HY.S wrote the manuscript; Z.G, R.S and DG.W revised the manuscript.; all authors reviewed and approved the manuscript for publication.

## FUNDING INFORMATION

This work was financially supported by the National Natural Science Foundation of China (Number 82071695 and 82,060,535), Young scientific and technological talent innovation project of Lanzhou (2023‐QN‐79), Fundamental Research Funds for the Central Universities (lzujbky‐2022‐it14), and the Open fund project of NHC Key Laboratory of Diagnosis and Therapy of Gastrointestinal Tumours (NLDTG2020006).

## CONFLICT OF INTEREST STATEMENT

The authors declare that they have no known competing financial interests or personal relationships that could have appeared to influence the work reported in this paper. All authors have read and approved the final manuscript.

## ETHICS STATEMENT

The animal study was reviewed and approved by Ethics Committee of Basic Medical School of Lanzhou University.

## Supporting information


**Data S1.** Supporting Information.

## Data Availability

The data that support the findings of this study are available from the corresponding author upon reasonable request.
