# Supplementary material for: Crotonylation of MCM6 enhances chemotherapeutics sensitivity of breast cancer via inducing DNA replication stress
Source: Cell Prolif. 2024 Oct 30;58(2):e13759. doi: 10.1111/cpr.13759 (PMC11839194; doi:10.1111/cpr.13759)
Supplement: Supplementary file 1 — Data S1. Supporting Information. [file CPR-58-e13759-s001.pdf]

**Supplementary Figure legends**

Supplement Figure 1. Pan-cancer analysis of MCM6

**A** Bulk tissue gene expression for MCM6 based on the GTEx database. **B** Expression of MCM6 in different clinical stages of pancancer. **C** Association of MCM6 expression with cancer and diseases, respectively.

Supplement Figure 2. Functional enrichment analysis of MCM6

**A** The coexpression network presents the coexpressed genes of MCM6. **B** Biofunctional sub-clusters extracted from the main PPI network from Metascape database. **C** GO/KEGG pathway enrichment for MCM6. **D** The GO enrichment based on MCM6 binding proteins using Coexpedia database.

Supplement Figure 3. Aberrant expression of MCM6 serves as a diagnostic biomarker among cancers

**A** Circular histogram of the receiver operating characteristic (ROC) curves of MCM6 in 27 cancers (AUC>0.5). **B** A forest plot of overall survival (OS) hazard ratios of MCM6 in 33 types of tumors. **C** A forest plot of progression free interval (PFI) hazard ratios of MCM6 in 33 types of tumors.

Supplement Figure 4. The association between MCM6 and breast cancer

**A** The visualization of institutions researching MCM6. **B** Visualization of the cocitation reference network for MCM6 research. **C** Keyword analysis of MCM6

from 2005 to 2023. **D** KEGG analysis of MCM6 co-expression genes in BRCA cohort. **E** Relative MCM6 mRNA expression across a panel of breast cancer cell lines. **F** Western blotting of MCM6 expression in human embryonic kidney cells (293T), melanoma cells (A2078 and A375), hepatocellular carcinoma cells (Hep-G2), and breast cancer cells (MDA-MC-231, T-47D, 4T1 and MCF7).

Supplement Figure 5. MCM6 knockdown induces cells apoptosis by inducing DNA replication stress

**A-C** MCF7 cells were transduced with siRNA against MCM6, following processed for immunofluorescence using γH2AX (**A**), Rad51 (**B**), and BRCA1 (**C**) antibodies. **D-E** Knockdown of MCM6 induced MCF7 cell apoptosis, as detected by TUNEL staining (**D**) and AnnexinV staining (**E**). **F** Western blot analysis of apoptosis-related protein Bax and Bcl-xl in breast cancer cell. Mean±SD, n=3. *$p < 0.05$, **$p < 0.01$, ***$p < 0.001$, vs. control.

Supplement Figure 6. NaCR-mediated crotonylation of MCM6 suppresses BRCA cells proliferation

**A** Bar graphs showing the distribution of the number of identified Kcr sites and proteins. **B** A table summarizing the top 10 proteins identified by this study. **C** Analysis of biological of upregulated Kcr proteins by GO enrichment. **D** Chromatin lysates of different time points of NaCR were isolated, and western blotting was performed using p-CHK2 antibodies in MCF7 and 4T1 cell.

Supplement Figure 7. Lysine crotonylation of MCM6 at K599 is involved in regulating DNA replication stress in breast cancer cells

**A** LC-MS/MS spectra of the crotonylation modified peptide. **B** Schematic diagram illustrating the 2D structure of MCM6. **C-D** Conserved domain analysis of MCM6 using the MEGA 5.1 (**C**) and WebLogo website (**D**). **E** The potential modified sites of MCM6 in the amino acid sequence. **F** MCM6 K599 site-specific mutagenesis was verified by next-generation sequencing.

Supplement Figure 8. MCM6-K599cr results in BRCA cells apoptosis due to severe DNA damage

**A** Scratch wound assay of 4T1 transfected with plasmid specific to MCM6-K599A and MCM6-K599Q. **B-D** MCF7 cells were transduced with plasmid against K599Q and K599A, following processed for immunofluorescence using γH2AX (**B**), BRCA1 (**C**), and Rad51 (**D**) antibodies. **E-F** Tranfection of MCM6-K599Q induced MCF7 cell apoptosis, as detected by TUNEL staining (**E**) and AnnexinV staining (**F**). **G** Western blot analysis of apoptosis-related protein Bax and Bcl-xl in breast cancer cell. Mean±SD, n=3. *$p < 0.05$, **$p < 0.01$, ***$p < 0.001$, vs. control.

Supplement Figure 9. Kaempferol is a potential inhibitor targeting SIRT7

**A** Prediction of ingredients targeting SIRT7 based on SymMap V2 and CTD databse. **B** Molecular docking using CB-dock 2 were performed between SIRT7 and

kaempferol.

Supplement Figure 10. Kaempferol as a potential regulator in breast cancer cells

**A-B** Disease ontology (DO) indicated kaempferol associated disease progression based on TCM, OMIM, and CTD database. **C-D** A network and histogram analyzed kaempferol-associated GO pathways enrichment.

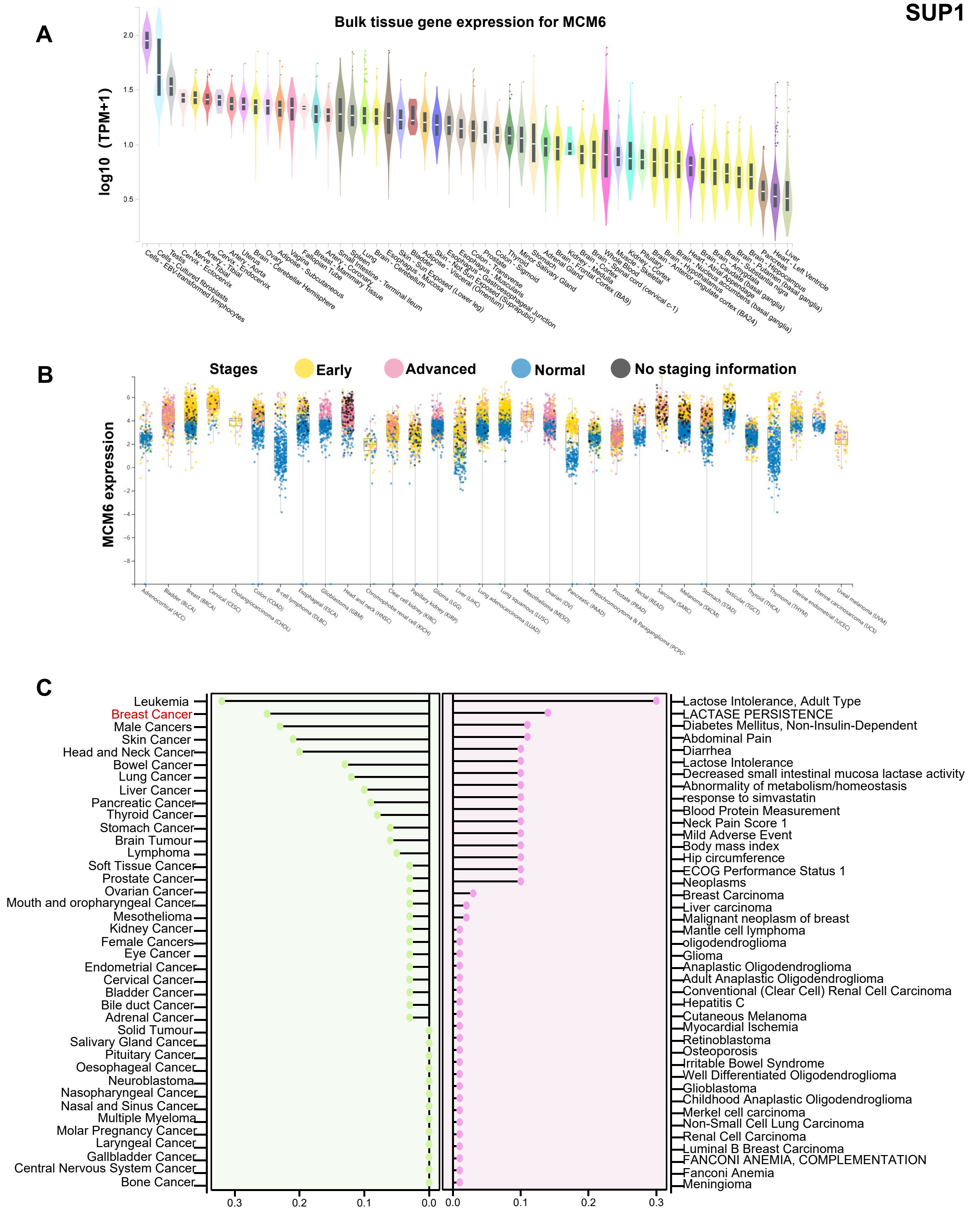
SUP1
A
Bulk tissue gene expression for MCM6
log10 (TPM+1)
2.0
1.5
1.0
0.5
Cells - EBV-transformed lymphocytes
Cells - Cultured fibroblasts
Testis
Cervix - Ectocervix
Nerve - Tibial
Artery - Tibial
Cervix - Endocervix
Artery - Aorta
Uterus
Brain - Cerebellar Hemisphere
Ovary
Adipose - Subcutaneous
Vagina
Fallopian Tube
Breast - Mammary Tissue
Artery - Coronary
Small Intestine - Terminal Ileum
Spleen
Lung
Brain - Cerebellum
Esophagus - Mucosa
Skin - Sun Exposed (Lower leg)
Bladder
Adipose - Visceral (Omentum)
Skin - Not Sun Exposed (Suprapubic)
Esophagus - Gastroesophageal Junction
Esophagus - Muscularis
Colon - Transverse
Prostate
Colon - Sigmoid
Thyroid
Minor Salivary Gland
Stomach
Adrenal Gland
Brain - Frontal Cortex (BA9)
Kidney - Medulla
Brain - Cortex
Brain - Spinal cord (cervical c-1)
Whole Blood
Muscle - Skeletal
Kidney - Cortex
Pituitary
Brain - Anterior cingulate cortex (BA24)
Brain - Hypothalamus
Heart - Atrial Appendage
Brain - Nucleus accumbens (basal ganglia)
Brain - Amygdala
Brain - Caudate (basal ganglia)
Brain - Substantia nigra
Brain - Hippocampus
Pancreas
Brain - Putamen (basal ganglia)
Heart - Left Ventricle
Liver
B
Stages
Early
Advanced
Normal
No staging information
MCM6 expression
6
4
2
0
-2
-4
-6
-8
Adrenocortical (ACC)
Bladder (BLCA)
Breast (BRCA)
Cervical (CESC)
Cholangiocarcinoma (CHOL)
Colon (COAD)
B-cell lymphoma (DLBC)
Esophageal (ESCA)
Glioblastoma (GBM)
Head and neck (HNSC)
Chromophobe renal cell (KICH)
Clear cell kidney (KIRC)
Papillary kidney (KIRP)
Glioma (LGG)
Liver (LIHC)
Lung adenocarcinoma (LUAD)
Lung squamous (LUSC)
Mesothelioma (MESO)
Ovarian (OV)
Pancreatic (PAAD)
Pheochromocytoma & Paraganglioma (PCPG)
Prostate (PRAD)
Rectal (READ)
Sarcoma (SARC)
Melanoma (SKCM)
Stomach (STAD)
Testicular (TGCT)
Thyroid (THCA)
Thymoma (THYM)
Uterine endometrial (UCEC)
Uterine carcinosarcoma (UCS)
Uveal melanoma (UVM)
C
Leukemia
Breast Cancer
Male Cancers
Skin Cancer
Head and Neck Cancer
Bowel Cancer
Lung Cancer
Liver Cancer
Pancreatic Cancer
Thyroid Cancer
Stomach Cancer
Brain Tumour
Lymphoma
Soft Tissue Cancer
Prostate Cancer
Ovarian Cancer
Mouth and oropharyngeal Cancer
Mesothelioma
Kidney Cancer
Female Cancers
Eye Cancer
Endometrial Cancer
Cervical Cancer
Bladder Cancer
Bile duct Cancer
Adrenal Cancer
Solid Tumour
Salivary Gland Cancer
Pituitary Cancer
Oesophageal Cancer
Neuroblastoma
Nasopharyngeal Cancer
Nasal and Sinus Cancer
Multiple Myeloma
Molar Pregnancy Cancer
Laryngeal Cancer
Gallbladder Cancer
Central Nervous System Cancer
Bone Cancer
0.3
0.2
0.1
0.0
0.0
0.1
0.2
0.3
Lactose Intolerance, Adult Type
LACTASE PERSISTENCE
Diabetes Mellitus, Non-Insulin-Dependent
Abdominal Pain
Diarrhea
Lactose Intolerance
Decreased small intestinal mucosa lactase activity
Abnormality of metabolism/homeostasis
response to simvastatin
Blood Protein Measurement
Neck Pain Score 1
Mild Adverse Event
Body mass index
Hip circumference
ECOG Performance Status 1
Neoplasms
Breast Carcinoma
Liver carcinoma
Malignant neoplasm of breast
Mantle cell lymphoma
oligodendroglioma
Glioma
Anaplastic Oligodendroglioma
Adult Anaplastic Oligodendroglioma
Conventional (Clear Cell) Renal Cell Carcinoma
Hepatitis C
Cutaneous Melanoma
Myocardial Ischemia
Retinoblastoma
Osteoporosis
Irritable Bowel Syndrome
Well Differentiated Oligodendroglioma
Glioblastoma
Childhood Anaplastic Oligodendroglioma
Merkel cell carcinoma
Non-Small Cell Lung Carcinoma
Renal Cell Carcinoma
Luminal B Breast Carcinoma
FANCONI ANEMIA, COMPLEMENTATION
Fanconi Anemia
Meningioma

SUP2

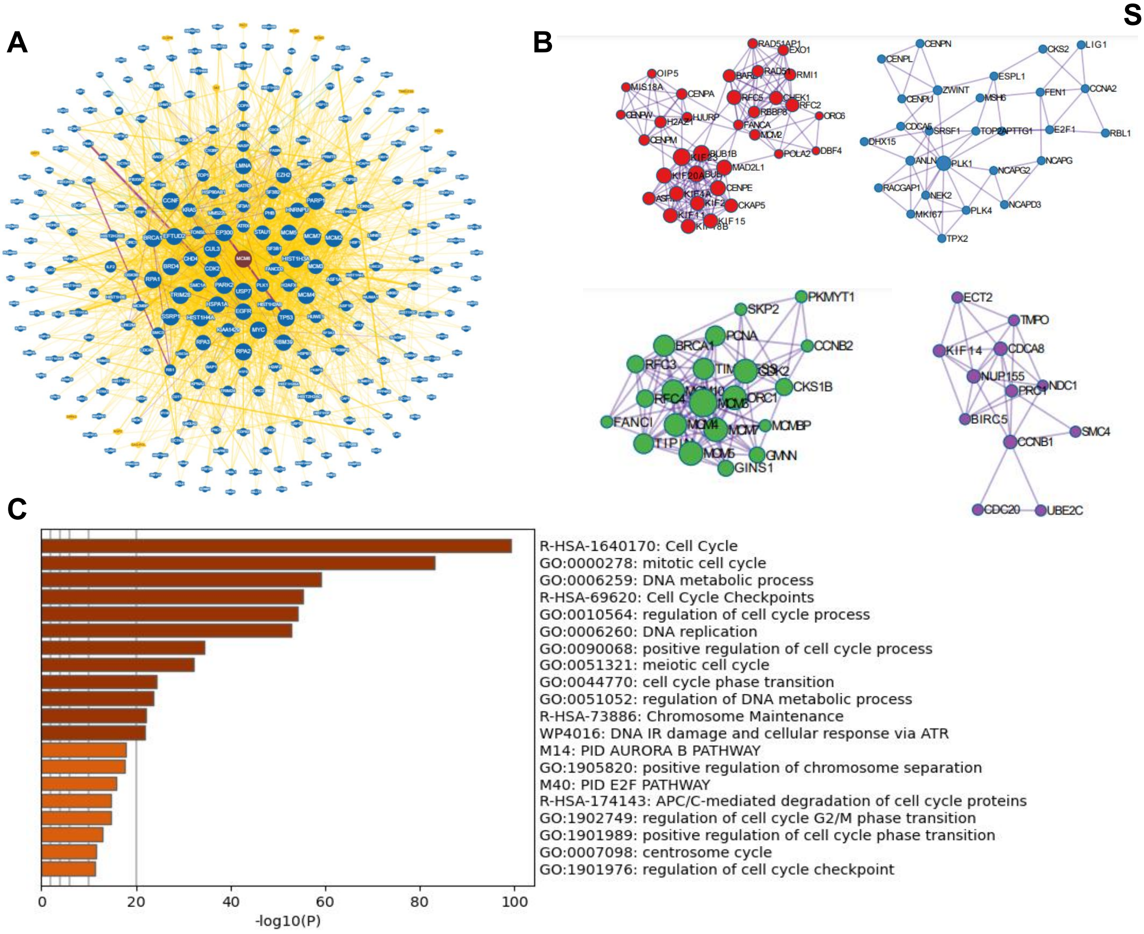


D

| Rank | GO Acc. and Desc. | *p*-value |
|---|---|---|
| 1 | GO:0006260-DNA replication | 3.25E-51 |
| 2 | GO:0007062-sister chromatid cohesion | 4.92E-43 |
| 3 | GO:0000082-G1/S transition of mitotic cell cycle | 1.37E-33 |
| 4 | GO:0006281-DNA repair | 1.50E-20 |
| 5 | GO:0000731-DNA synthesis involved in DNA repair | 1.10E-18 |
| 6 | GO:0006271-DNA strand elongation involved in DNA replication | 8.81E-16 |
| 7 | GO:0000086-G2/M transition of mitotic cell cycle | 8.81E-16 |
| 8 | GO:0006297-nucleotide-excision repair, DNA gap filling | 4.59E-12 |
| 9 | GO:0042769-DNA damage response, detection of DNA damage | 5.86E-12 |
| 10 | GO:0051436-negative regulation of ubiquitin-protein ligase activity involved in mitotic cell cycle | 1.59E-11 |

SUP3

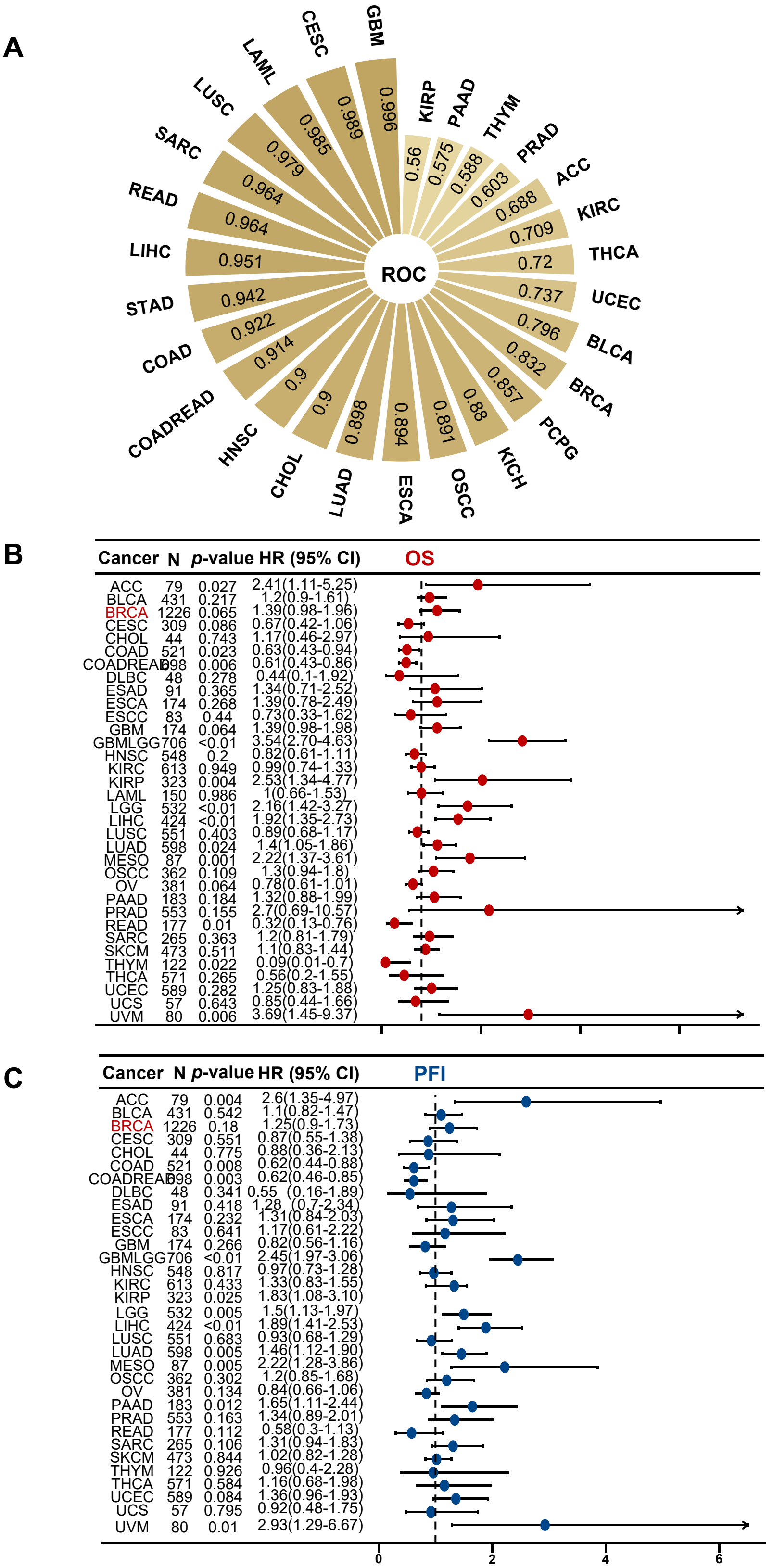
A
ROC
GBM 0.996
CESC 0.989
LAML 0.985
LUSC 0.979
SARC 0.964
READ 0.964
LIHC 0.951
STAD 0.942
COAD 0.922
COADREAD 0.914
HNSC 0.9
CHOL 0.9
LUAD 0.898
ESCA 0.894
OSCC 0.891
KICH 0.88
PCPG 0.857
BRCA 0.832
BLCA 0.796
UCEC 0.737
THCA 0.72
KIRC 0.709
ACC 0.688
PRAD 0.603
THYM 0.588
PAAD 0.575
KIRP 0.56
B
Cancer N p-value HR (95% CI) OS
ACC 79 0.027 2.41(1.11-5.25)
BLCA 431 0.217 1.2(0.9-1.61)
BRCA 1226 0.065 1.39(0.98-1.96)
CESC 309 0.086 0.67(0.42-1.06)
CHOL 44 0.743 1.17(0.46-2.97)
COAD 521 0.023 0.63(0.43-0.94)
COADREAD698 0.006 0.61(0.43-0.86)
DLBC 48 0.278 0.44(0.1-1.92)
ESAD 91 0.365 1.34(0.71-2.52)
ESCA 174 0.268 1.39(0.78-2.49)
ESCC 83 0.44 0.73(0.33-1.62)
GBM 174 0.064 1.39(0.98-1.98)
GBMLGG706 <0.01 3.54(2.70-4.63)
HNSC 548 0.2 0.82(0.61-1.11)
KIRC 613 0.949 0.99(0.74-1.33)
KIRP 323 0.004 2.53(1.34-4.77)
LAML 150 0.986 1(0.66-1.53)
LGG 532 <0.01 2.16(1.42-3.27)
LIHC 424 <0.01 1.92(1.35-2.73)
LUSC 551 0.403 0.89(0.68-1.17)
LUAD 598 0.024 1.4(1.05-1.86)
MESO 87 0.001 2.22(1.37-3.61)
OSCC 362 0.109 1.3(0.94-1.8)
OV 381 0.064 0.78(0.61-1.01)
PAAD 183 0.184 1.32(0.88-1.99)
PRAD 553 0.155 2.7(0.69-10.57)
READ 177 0.01 0.32(0.13-0.76)
SARC 265 0.363 1.2(0.81-1.79)
SKCM 473 0.511 1.1(0.83-1.44)
THYM 122 0.022 0.09(0.01-0.7)
THCA 571 0.265 0.56(0.2-1.55)
UCEC 589 0.282 1.25(0.83-1.88)
UCS 57 0.643 0.85(0.44-1.66)
UVM 80 0.006 3.69(1.45-9.37)
C
Cancer N p-value HR (95% CI) PFI
ACC 79 0.004 2.6(1.35-4.97)
BLCA 431 0.542 1.1(0.82-1.47)
BRCA 1226 0.18 1.25(0.9-1.73)
CESC 309 0.551 0.87(0.55-1.38)
CHOL 44 0.775 0.88(0.36-2.13)
COAD 521 0.008 0.62(0.44-0.88)
COADREAD698 0.003 0.62(0.46-0.85)
DLBC 48 0.341 0.55 (0.16-1.89)
ESAD 91 0.418 1.28 (0.7-2.34)
ESCA 174 0.232 1.31(0.84-2.03)
ESCC 83 0.641 1.17(0.61-2.22)
GBM 174 0.266 0.82(0.56-1.16)
GBMLGG706 <0.01 2.45(1.97-3.06)
HNSC 548 0.817 0.97(0.73-1.28)
KIRC 613 0.433 1.33(0.83-1.55)
KIRP 323 0.025 1.83(1.08-3.10)
LGG 532 0.005 1.5(1.13-1.97)
LIHC 424 <0.01 1.89(1.41-2.53)
LUSC 551 0.683 0.93(0.68-1.29)
LUAD 598 0.005 1.46(1.12-1.90)
MESO 87 0.005 2.22(1.28-3.86)
OSCC 362 0.302 1.2(0.85-1.68)
OV 381 0.134 0.84(0.66-1.06)
PAAD 183 0.012 1.65(1.11-2.44)
PRAD 553 0.163 1.34(0.89-2.01)
READ 177 0.112 0.58(0.3-1.13)
SARC 265 0.106 1.31(0.94-1.83)
SKCM 473 0.844 1.02(0.82-1.28)
THYM 122 0.926 0.96(0.4-2.28)
THCA 571 0.584 1.16(0.68-1.98)
UCEC 589 0.084 1.36(0.96-1.93)
UCS 57 0.795 0.92(0.48-1.75)
UVM 80 0.01 2.93(1.29-6.67)
0
2
4
6

SUP4

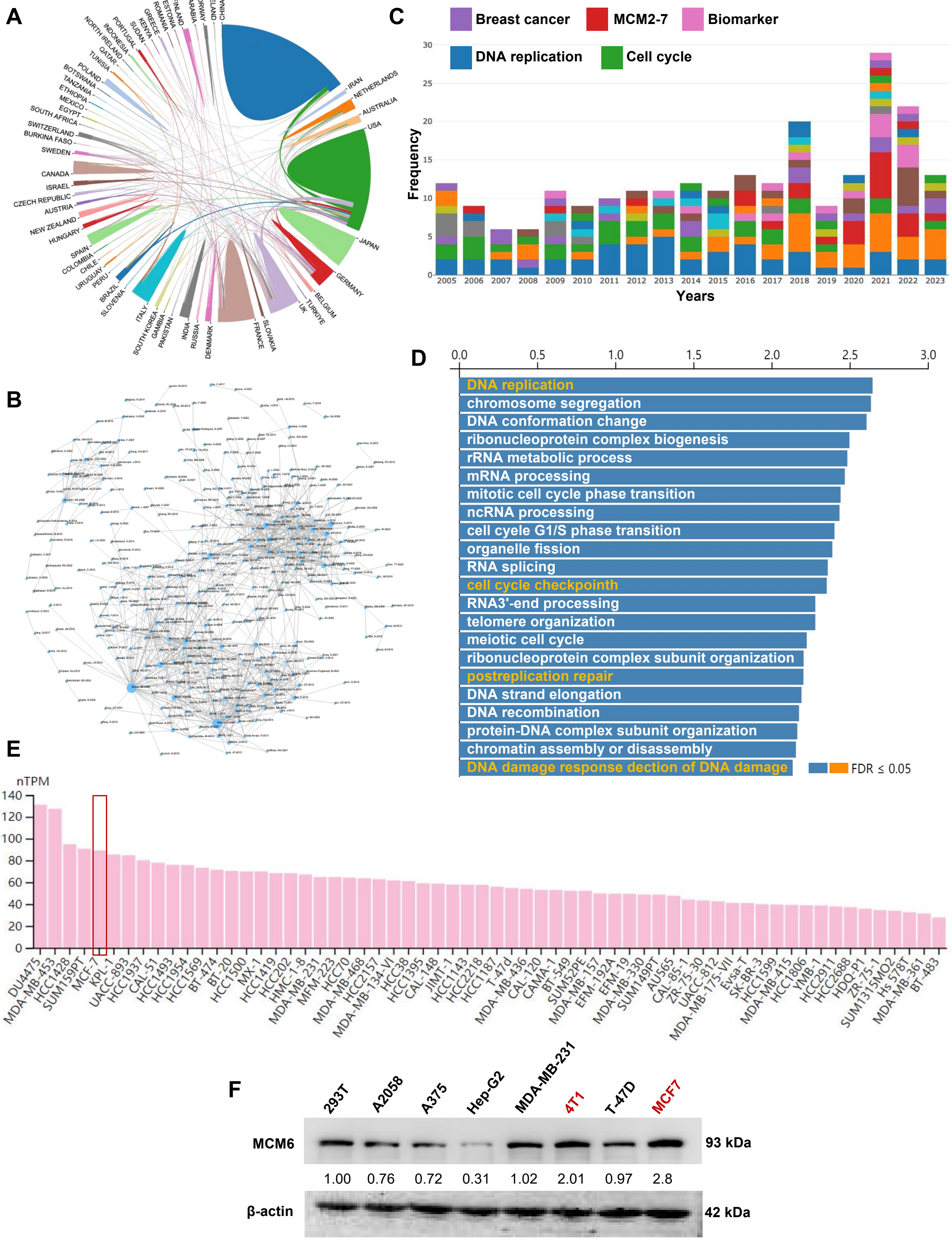
A
CHINA
IRELAND
NORWAY
SAUDI ARABIA
FINLAND
ESTONIA
ROMANIA
GREECE
KENYA
SUDAN
PORTUGAL
INDONESIA
NORTH IRELAND
QATAR
TUNISIA
POLAND
BOTSWANA
TANZANIA
ETHIOPIA
MEXICO
EGYPT
SOUTH AFRICA
SWITZERLAND
BURKINA FASO
SWEDEN
CANADA
ISRAEL
CZECH REPUBLIC
AUSTRIA
NEW ZEALAND
HUNGARY
SPAIN
COLOMBIA
CHILE
URUGUAY
PERU
BRAZIL
SLOVENIA
ITALY
SOUTH KOREA
GAMBIA
PAKISTAN
INDIA
RUSSIA
DENMARK
FRANCE
SLOVAKIA
UK
TURKIYE
BELGIUM
GERMANY
JAPAN
USA
AUSTRALIA
NETHERLANDS
IRAN
B
C
Breast cancer
MCM2-7
Biomarker
DNA replication
Cell cycle
Frequency
0
5
10
15
20
25
30
2005
2006
2007
2008
2009
2010
2011
2012
2013
2014
2015
2016
2017
2018
2019
2020
2021
2022
2023
Years
D
0.0
0.5
1.0
1.5
2.0
2.5
3.0
DNA replication
chromosome segregation
DNA conformation change
ribonucleoprotein complex biogenesis
rRNA metabolic process
mRNA processing
mitotic cell cycle phase transition
ncRNA processing
cell cyele G1/S phase transition
organelle fission
RNA splicing
cell cycle checkpointh
RNA3'-end processing
telomere organization
meiotic cell cycle
ribonucleoprotein complex subunit organization
postreplication repair
DNA strand elongation
DNA recombination
protein-DNA complex subunit organization
chromatin assembly or disassembly
DNA damage response dection of DNA damage
FDR ≤ 0.05
E
nTPM
0
20
40
60
80
100
120
140
DU4475
MDA-MB-453
HCC1428
SUM159PT
MCF-7
KPL-1
UACC-893
HCC1937
CAL-51
HCC1493
HCC1954
HCC1569
BT-474
BT-20
HCC1500
MX-1
HCC1419
HCC202
HMC-1-8
MDA-MB-231
MFM-223
HCC70
MDA-MB-468
HCC2157
MDA-MB-134-VI
HCC38
HCC1395
CAL-148
JIMT-1
HCC1143
HCC2218
HCC1187
T-47d
MDA-MB-436
CAL-120
CAMA-1
BT-549
SUM52PE
MDA-MB-157
EFM-192A
EFM-19
MDA-MB-330
SUM149PT
AU565
CAL-85-1
ZR-75-30
UACC-812
MDA-MB-175-VII
Evsa-T
SK-BR-3
HCC1599
MDA-MB-415
HCC1806
YMB-1
HCC2911
HCC2688
HDQ-P1
ZR-75-1
SUM1315MO2
Hs 578T
MDA-MB-361
BT-483
F
293T
A2058
A375
Hep-G2
MDA-MB-231
4T1
T-47D
MCF7
MCM6
93 kDa
1.00
0.76
0.72
0.31
1.02
2.01
0.97
2.8
β-actin
42 kDa

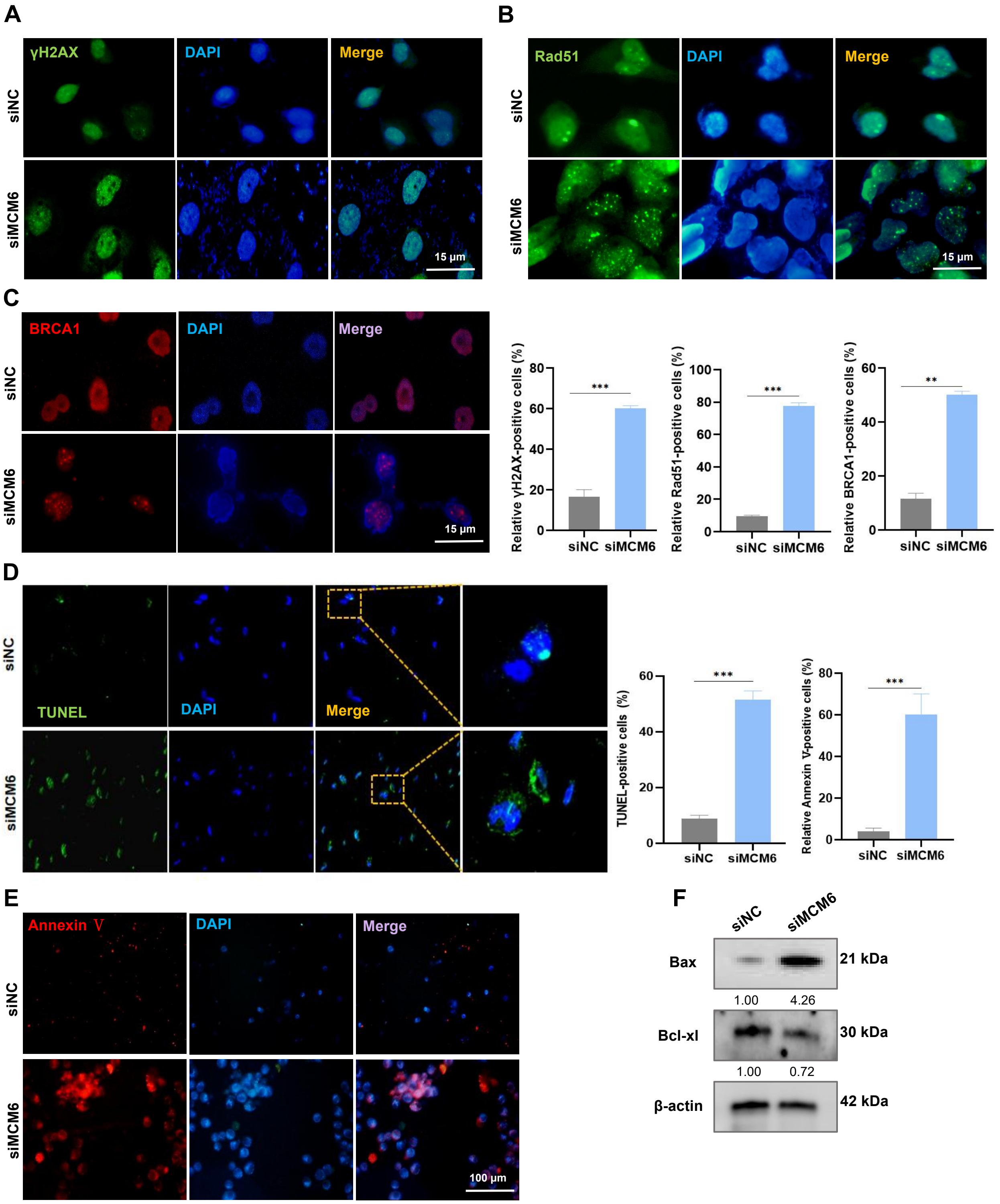
A
γH2AX
DAPI
Merge
siNC
siMCM6
15 μm
B
Rad51
DAPI
Merge
siNC
siMCM6
15 μm
C
BRCA1
DAPI
Merge
siNC
siMCM6
15 μm
Relative γH2AX-positive cells (%)
Relative Rad51-positive cells (%)
Relative BRCA1-positive cells (%)
***
**
siNC siMCM6
D
TUNEL
DAPI
Merge
siNC
siMCM6
TUNEL-positive cells (%)
Relative Annexin V-positive cells (%)
E
Annexin V
DAPI
Merge
siNC
siMCM6
100 μm
F
siNC siMCM6
Bax
21 kDa
1.00 4.26
Bcl-xl
30 kDa
1.00 0.72
β-actin
42 kDa

SUP6

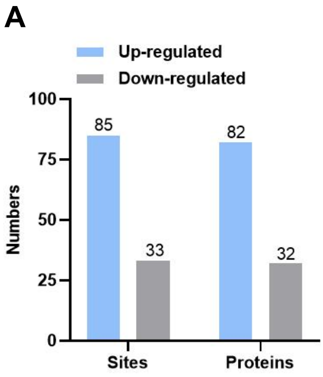


B

| Gene | Regulated type |
|---|---|
| MCM6 | Up |
| PRDX1 | Up |
| TFAM | Up |
| PGK1 | Up |
| SF3B1 | Down |
| PDCD6IP | Up |
| IKBIP | Up |
| SLC25A5 | Up |
| RTN3 | Up |
| MDH1 | Up |

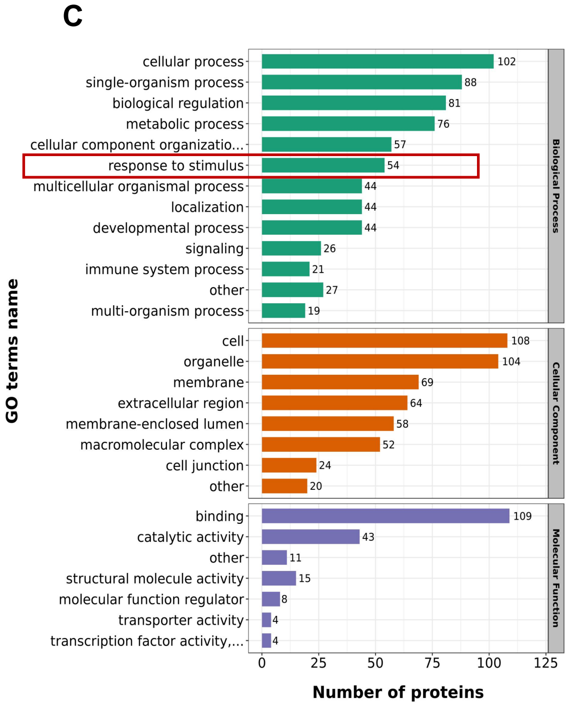


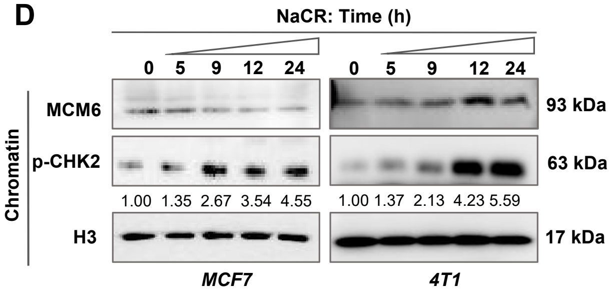

SUP7

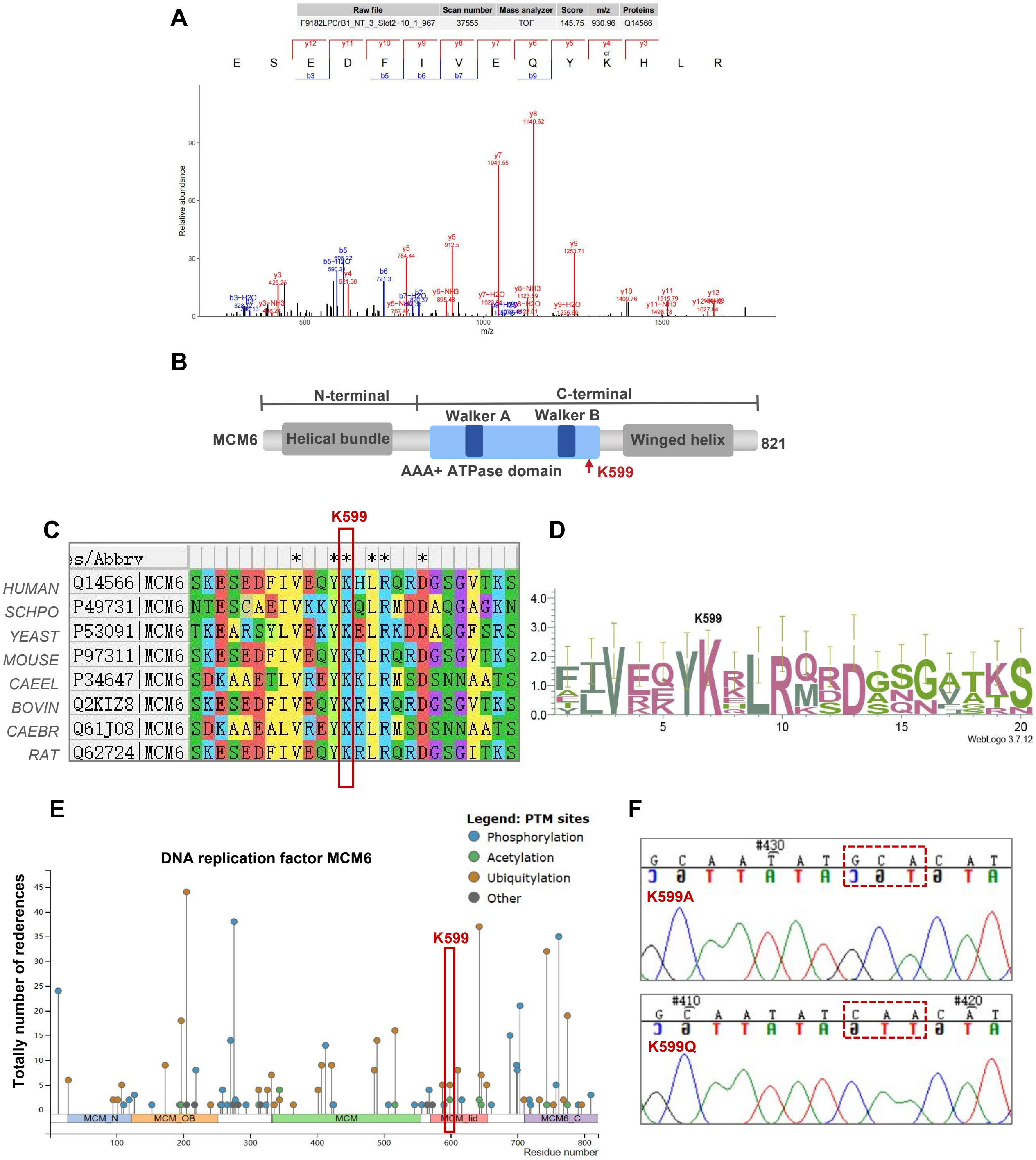
A
Raw file | Scan number | Mass analyzer | Score | m/z | Proteins
F9182LPCrB1_NT_3_Slot2-10_1_967 | 37555 | TOF | 145.75 | 930.96 | Q14566
E S E D F I V E Q Y K H L R
Relative abundance
m/z
B
N-terminal
C-terminal
Walker A
Walker B
MCM6
Helical bundle
Winged helix
821
AAA+ ATPase domain
K599
C
K599
HUMAN Q14566|MCM6 SKESEDFIVEQYKHLRQRDGSGVTKS
SCHPO P49731|MCM6 NTESCAEIVKKYKQLRMDDAQGAGKN
YEAST P53091|MCM6 TKEARSYLVEKYKELRKDDAQGFSRS
MOUSE P97311|MCM6 SKESEDFIVEQYKRLRQRDGSGVTKS
CAEEL P34647|MCM6 SDKAAETLVREYKKLRMSDSNNAATS
BOVIN Q2KIZ8|MCM6 SKESEDFIVEQYKRLRQRDGSGVTKS
CAEBR Q61J08|MCM6 SDKAAEALVREYKKLRMSDSNNAATS
RAT Q62724|MCM6 SKESEDFIVEQYKRLRQRDGSGITKS
D
K599
WebLogo 3.7.12
E
DNA replication factor MCM6
Legend: PTM sites
Phosphorylation
Acetylation
Ubiquitylation
Other
K599
Totally number of rederences
MCM_N
MCM_OB
MCM
MCM_lid
MCM6_C
Residue number
F
K599A
K599Q

SUP8

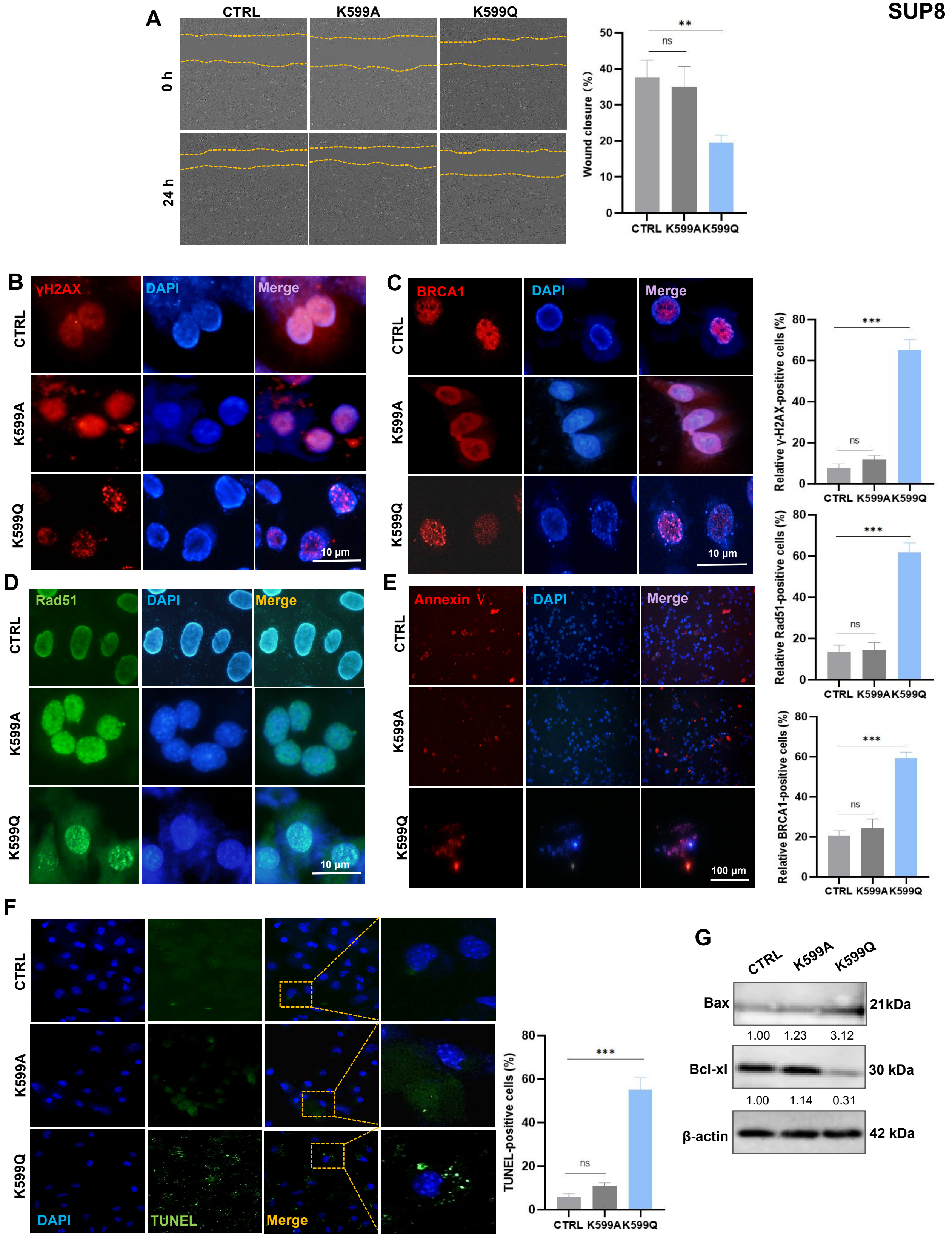
A
CTRL
K599A
K599Q
0 h
24 h
Wound closure (%)
ns
**
B
γH2AX
DAPI
Merge
10 μm
C
BRCA1
10 μm
Relative γ-H2AX-positive cells (%)
***
Relative Rad51-positive cells (%)
Relative BRCA1-positive cells (%)
D
Rad51
E
Annexin V
100 μm
F
TUNEL
TUNEL-positive cells (%)
G
Bax
21kDa
1.00
1.23
3.12
Bcl-xl
30 kDa
1.00
1.14
0.31
β-actin
42 kDa

A

| Ingredient | Structure | Evidence score |
|---|---|---|
| **Melatonin** | 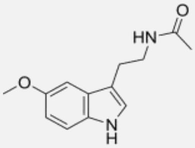 | **3.5** |
| **Nicotinamide** | 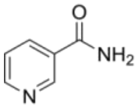 | **4** |
| **Astaxanthin** | 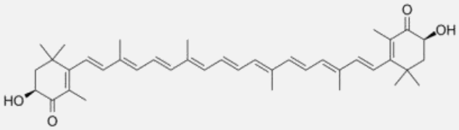 | **4** |
| **Kaempferol** | 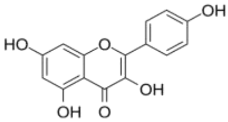 | **3.5** |
| **Resveratrol** | 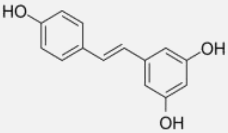 | **4** |
| **Quercetin** | 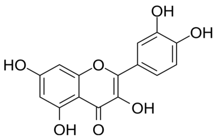 | **3.5** |

**B**

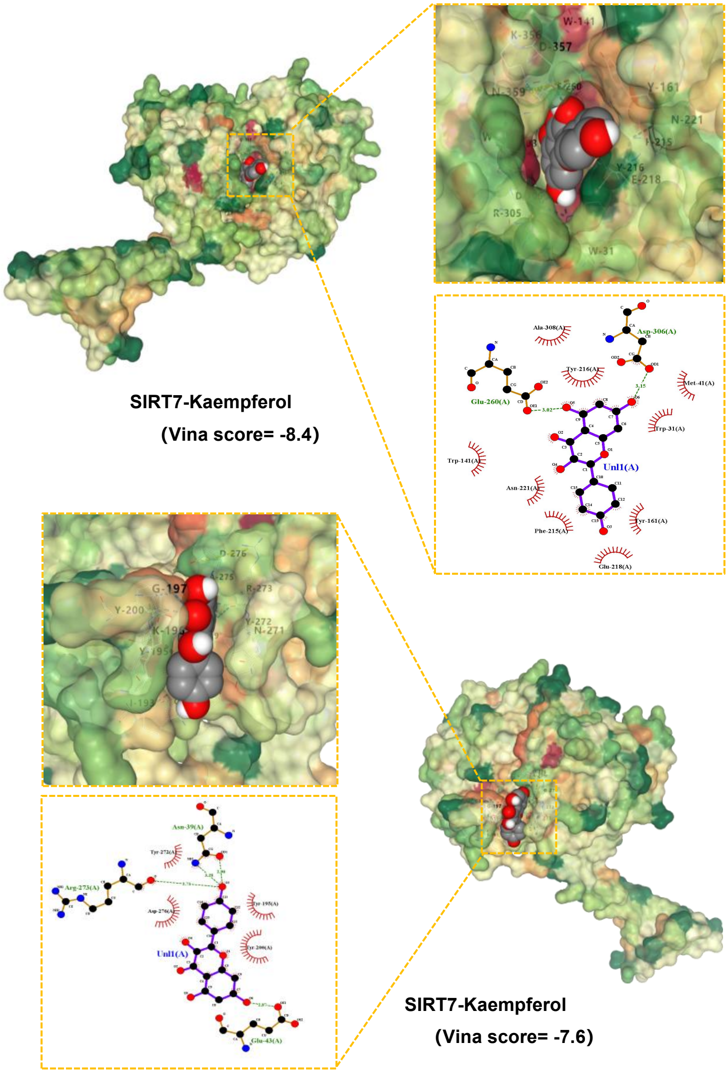
W-141
K-356
D-357
N-359
Y-161
N-221
F-215
Y-216
E-218
R-305
W-31
Ala-308(A)
Asp-306(A)
Tyr-216(A)
Met-41(A)
Glu-260(A)
3.02
3.15
Trp-31(A)
Trp-141(A)
Unl1(A)
Asn-221(A)
Tyr-161(A)
Phe-215(A)
Glu-218(A)
SIRT7-Kaempferol
(Vina score= -8.4)
D-276
A-275
G-197
R-273
Y-200
Y-272
N-271
Y-195
Asn-39(A)
Tyr-272(A)
Arg-273(A)
Asp-276(A)
Tyr-195(A)
Tyr-200(A)
Unl1(A)
Glu-43(A)
SIRT7-Kaempferol
(Vina score= -7.6)

SUP10

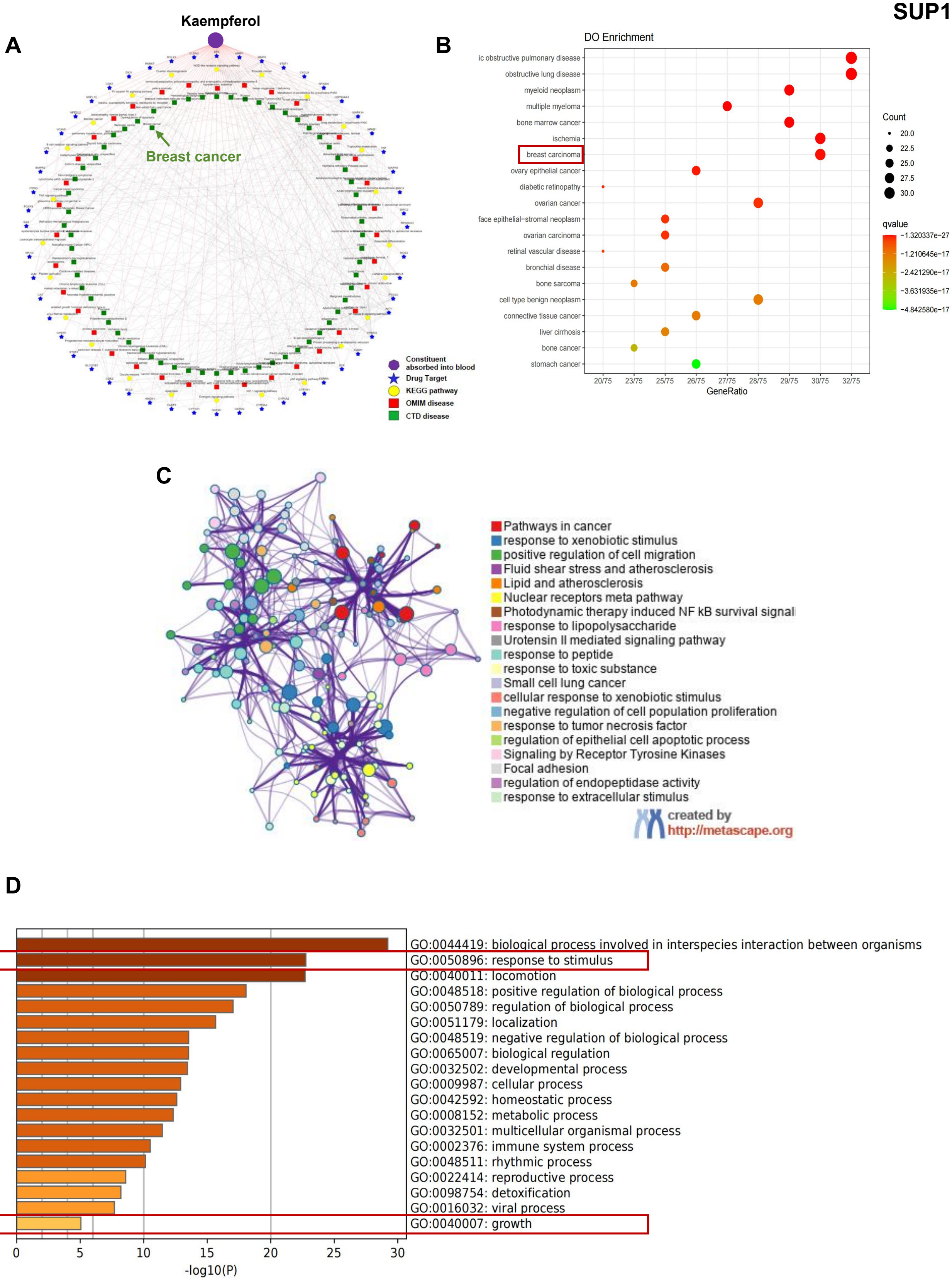

A
Kaempferol
Breast cancer
Constituent absorbed into blood
Drug Target
KEGG pathway
OMIM disease
CTD disease
B
DO Enrichment
ic obstructive pulmonary disease
obstructive lung disease
myeloid neoplasm
multiple myeloma
bone marrow cancer
ischemia
breast carcinoma
ovary epithelial cancer
diabetic retinopathy
ovarian cancer
face epithelial−stromal neoplasm
ovarian carcinoma
retinal vascular disease
bronchial disease
bone sarcoma
cell type benign neoplasm
connective tissue cancer
liver cirrhosis
bone cancer
stomach cancer
20/75
23/75
25/75
26/75
27/75
28/75
29/75
30/75
32/75
GeneRatio
Count
20.0
22.5
25.0
27.5
30.0
qvalue
−1.320337e−27
−1.210645e−17
−2.421290e−17
−3.631935e−17
−4.842580e−17
C
Pathways in cancer
response to xenobiotic stimulus
positive regulation of cell migration
Fluid shear stress and atherosclerosis
Lipid and atherosclerosis
Nuclear receptors meta pathway
Photodynamic therapy induced NF kB survival signal
response to lipopolysaccharide
Urotensin II mediated signaling pathway
response to peptide
response to toxic substance
Small cell lung cancer
cellular response to xenobiotic stimulus
negative regulation of cell population proliferation
response to tumor necrosis factor
regulation of epithelial cell apoptotic process
Signaling by Receptor Tyrosine Kinases
Focal adhesion
regulation of endopeptidase activity
response to extracellular stimulus
created by
http://metascape.org
D
GO:0044419: biological process involved in interspecies interaction between organisms
GO:0050896: response to stimulus
GO:0040011: locomotion
GO:0048518: positive regulation of biological process
GO:0050789: regulation of biological process
GO:0051179: localization
GO:0048519: negative regulation of biological process
GO:0065007: biological regulation
GO:0032502: developmental process
GO:0009987: cellular process
GO:0042592: homeostatic process
GO:0008152: metabolic process
GO:0032501: multicellular organismal process
GO:0002376: immune system process
GO:0048511: rhythmic process
GO:0022414: reproductive process
GO:0098754: detoxification
GO:0016032: viral process
GO:0040007: growth
0
5
10
15
20
25
30
-log10(P)
